# Multi-Targeted Anticancer Activity of Imidazolate Phosphane Gold(I) Compounds by Inhibition of DHFR and TrxR in Breast Cancer Cells

**DOI:** 10.3389/fchem.2020.602845

**Published:** 2021-01-11

**Authors:** Rossana Galassi, Lorenzo Luciani, Valentina Gambini, Silvia Vincenzetti, Giulio Lupidi, Augusto Amici, Cristina Marchini, Junbiao Wang, Stefania Pucciarelli

**Affiliations:** ^1^School of Science and Technology, University of Camerino, Camerino, Italy; ^2^School of Biosciences and Veterinary Medicine, University of Camerino, Camerino, Italy; ^3^School of Drugs and Health Products Sciences, University of Camerino, Camerino, Italy

**Keywords:** gold, enzyme inhibition, anticancer agents, metal based drug, gold phosphane compounds, DiHydroFolateReductase, thioredoxinReductase, breast cancer

## Abstract

A class of phosphane gold(I) compounds, made of azoles and phosphane ligands, was evaluated for a screening on the regards of Breast Cancer cell panels (BC). The compounds possess N-Au-P or Cl-Au-P bonds around the central metal, and they differ for the presence of aprotic or protic polar groups in the azoles and/or the phosphane moieties to tune their hydrophilicity. Among the six candidates, only the compounds having the P-Au-N environment and not displaying neither the hydroxyl nor carboxyl groups in the ligands were found active. The compounds were screened by MTT tests in SKBR3, A17, and MDA-MB231 cancer cells, and two compounds (namely the 4,5-dicyano-imidazolate-1yl-gold(I)-(triphenylphosphane, 5, and 4,5-dichloro-imidazolate-1yl-gold(I)-triphenylphosphane, 6) were found very cytotoxic, with the most active with an IC_50_ value of 3.46 μM in MDA-MB231 cells. By performing enzymatic assays in the treated cells lysates, the residual enzymatic activity of dihydrofolate reductase (DHFR) has been measured after cell treatment for 4 or 12 h in comparison with control cells. Upon 12 h of treatment, the activity of DHFR was significantly reduced in both SKBR3 and A17 cells by compounds 5 and 6, but not in human MDA-MB231 cells; interestingly, it was found remarkably high after 4 h of treatment, revealing a time dependence for the DHFR enzymatic assays. The DHFR inhibition data have been compared to those for the thioredoxin reductase (TrxR), the most recognized molecular target for gold compounds. For this latter, similar residual activities (i.e., 37 and 49% for the match of SKBR3 cells and compound 5 or 6, respectively) were found. Binding studies on the regards of ct-DNA (calf-thymus-DNA) and of plasma transporters proteins, such as BSA (bovine serum albumin) and ATF (apo transferrin), were performed. As expected for gold compounds, the data support strong binding to proteins (K_sv_ values range: 1.51 ÷ 2.46 × 10^4^ M^−1^) and a weaker interaction with ct-DNA's minor groove (K_sv_ values range: 1.55 ÷ 6.12 × 10^3^ M^−1^).

## Introduction

Breast Cancer (BC) is the second most frequent cancer worldwide and, by far, the most recurrent cancer among the female gender with the esteem of 2.1 million new cases detected in 2018 (25% of all cancers) (Bray et al., 2018). The term breast cancer expresses not a single disease but it includes four major molecular subtypes (Luminal A, Luminal B, HER2-positive and Basal Like Breast Cancer, BLBC), whose classification is based on the expression of hormone receptors (estrogen receptors (ER), and progesterone receptors (PR)) and Human Epidermal growth factor Receptor 2 (HER2) (Burstein, 2005). Optimal therapy for each patient depends on the tumor subtype. Luminal A (ER-positive (+) and PR+) and Luminal B (ER+, PR+ and HER2+) may benefit from endocrine therapy. HER2 overexpression is associated with poor prognosis, but HER2+ BC patients can be treated with HER2-targeted therapies, such as the monoclonal antibodies trastuzumab and pertuzumab, the antibody-drug conjugate trastuzumab emtansine (T-DM1), and the tyrosine kinase inhibitor lapatinib, alone or in combination with chemotherapy (see Table 1). These targeted treatments improve patient overall survival but drug resistance mechanisms often compromise their long-term effectiveness and most patients eventually relapse. Among BC subtypes, BLBC is the most aggressive. No targeted therapies are currently available for BLBC as it lacks the expression of both hormone receptors and HER2 (Yao et al., 2016; Waks and Winer, 2019). Thus, BLBC is treated with a combination of surgery, radiotherapy and chemotherapy (Schwentner et al., 2012; Nakai et al., 2016; Zheng et al., 2018), that is often not successful against metastatic BLBC. BC chemotherapy consists of a mixture of drugs (Fisher et al., 1990) that includes metal based compounds such as carboplatin or analogs (Silver et al., 2010), and alkaloids, such as taxanes (Waks and Winer, 2019). All these anticancer drugs are very effective but the intervention of serious side effects and/or the high incidence of drug resistance phenomena triggers the research of new medications (Correia et al., 2018).

**Table 1:** Current therapeutic approaches to BC subtypes (adapted from Waks and Winer, 2019).

**Breast cancer receptor subtype**	**Therapeutic approach**		**Additional notes**
	**First line**	**Later lines of therapy**	
Hormone receptor positive (HR+) and *ERBB2-*	Aromatase inhibitor plus CDK4/6 inhibitora ORR = 53–59%	Hormonal and/or targeted therapyIf resistant to multiple lines of hormonal therapy, transition to single-agent chemotherapy.	Premenopausal patients with HR+ metastatic BC should undergo treatment to achieve hormonal and/or targeted therapy or surgical menopause.
*ERBB2*+	Taxane + trastuzumab + pertuzumab ORR^◦^= 80% Selected patients with HR+/*ERBB2*+ disease can receive endocrine therapy plus *ERBB2*-targeted therapy	ERBB2-targeted agent plus chemotherapy or endocrine therapy if HR+ Trastuzumab + chemotherapy Trastuzumab + endocrine therapy Lapatinib + capecitabine	Very common brain metastases may be treated with both local and systemic therapies.
Triple-negative	Single-agent chemotherapy Taxane ORR = 36% Platinum ORR rate = 31% Anthracycline	Single-agent chemotherapy^*^ Capecitabine Eribulin Vinorelbine Gemcitabine Olaparib or talazoparib (if germline *BRCA1/2* mutation)	There is no single recommended first-line chemotherapy regimen.

◦ *ORR, overall response rate*.

* *Other agents not administered in initial lines are also acceptable options*.

**Scheme 1:**
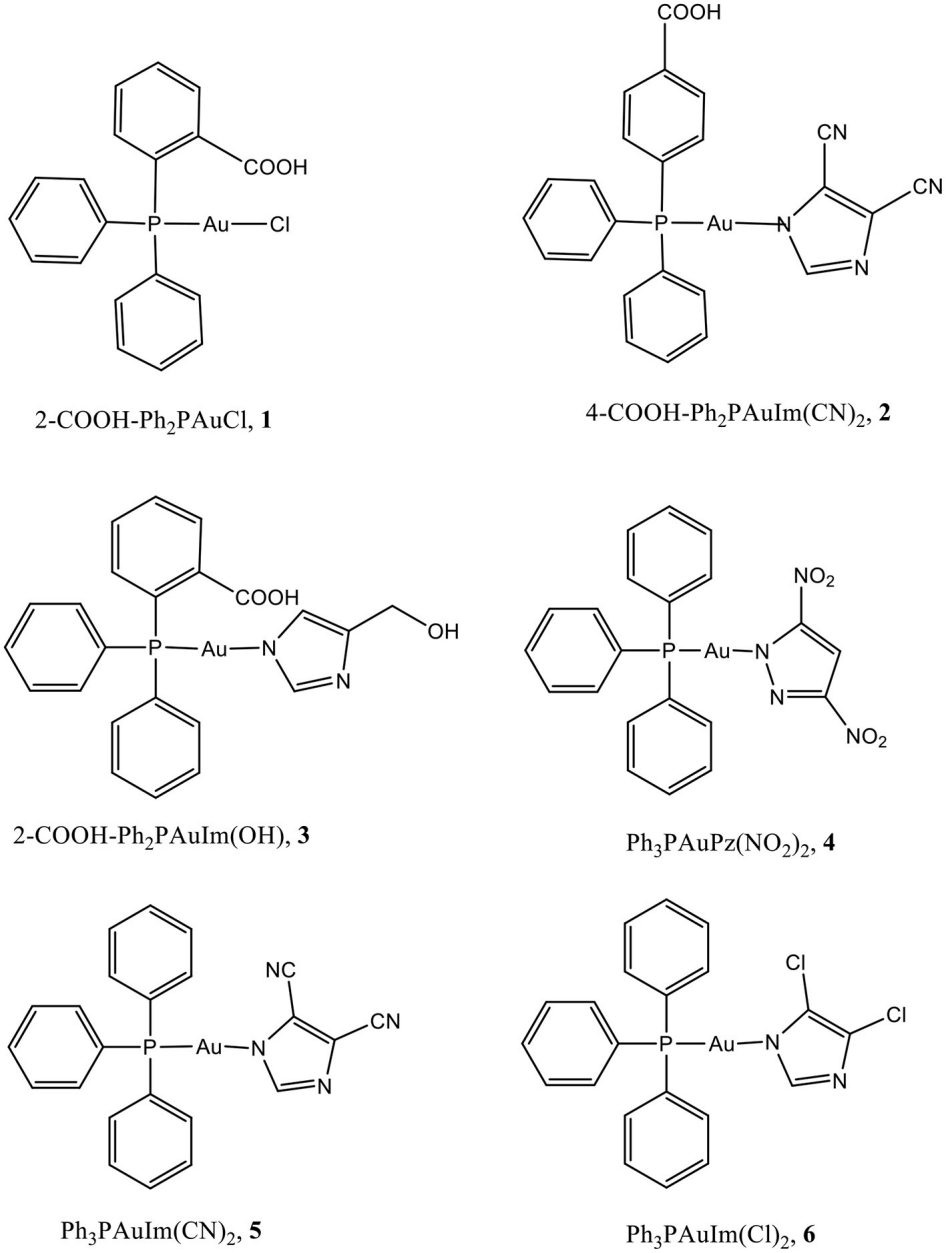
Sketch of the molecular structures considered in this work.

As concern the metal based drugs, in alternative to platin(II) compounds a large variety of gold(I) (Nobili et al., 2010; Berners-Price and Filipovska, 2011; Bertrand and Casini, 2014) and gold(III) compounds (Nobili et al., 2010; Che and Sun, 2011; da Silva Maia et al., 2014; Zou et al., 2015) have been considered; the attention on phosphane gold(I) compounds takes its origin from a renewed interest in Auranofin, an old antirheumatic drug made of an apolar head of triethylphosphane and a tetraacetyl-thiosugar polar tail, discovered to be a potent anticancer agent mainly acting by the inhibition of ThioRedoxin Reductase enzyme (TrxR) (Cui et al., 2020; Raninga et al., 2020). In general, lipophilic phosphane gold(I) compounds are characterized by remarkable antiproliferative properties (Kim et al., 2019), optimal cellular uptake depending on the molecule hydro/lipophilic balance (McKeage et al., 2000; Liu et al., 2008; Scheffler et al., 2010) and protein binding (Messori et al., 2014; Kim et al., 2019), on the other hand, water soluble highly hydrophilic phosphane metal compounds showed appreciable cytotoxic activity on the regard of MCF-7 BC cells (Santini et al., 2011). Recently, our group has found that azolate/phosphane gold(I) compounds are active on the regards of many panels of cancer lines (Galassi et al., 2012) and they also inhibit TrxR, with Ki in the nanomolar range. The azolate/phosphane gold(I) compounds retain their activity also *in vivo*, and two compounds 4,5-dicyano-imidazolate-1yl-gold(I)-(triphenylphosphane) or 4,5-dichloro-imidazolate-1yl-gold(I)-(triphenylphosphane), were found to contrast BLBC tumors transplanted in syngeneic mice; these compounds were found much more active than cisplatin, one of the most used metal based chemotherapy applied in BC, displaying fewer side effects and being in general more tolerated by the mice (Gambini et al., 2018). By considering these promising findings and as prosecution of this work, some of the previously characterized gold(I) compounds and additional new compounds were used in the present study (Scheme 1). In particular, all the compounds contain the N-Au-P bonds, leaving apart compound **1** which contains P-Au-Cl bonds; in addition, compounds **2** and **3** contain hydrophilic groups such as OH or COOH in the azoles and/or phosphane groups to evaluate whether the presence of polar, protic groups in the molecular structure affects the cytotoxic activity in different BC cell lines (Santini et al., 2011); compound **4** contains a different 3,5-disubstituted polar azole, the 3,5-dinitropyrazolate, and compounds **5** and **6** are the ones already studied and resulted to be effective as anticancer drugs *in vivo* (Gambini et al., 2018).

**Figure 1:**
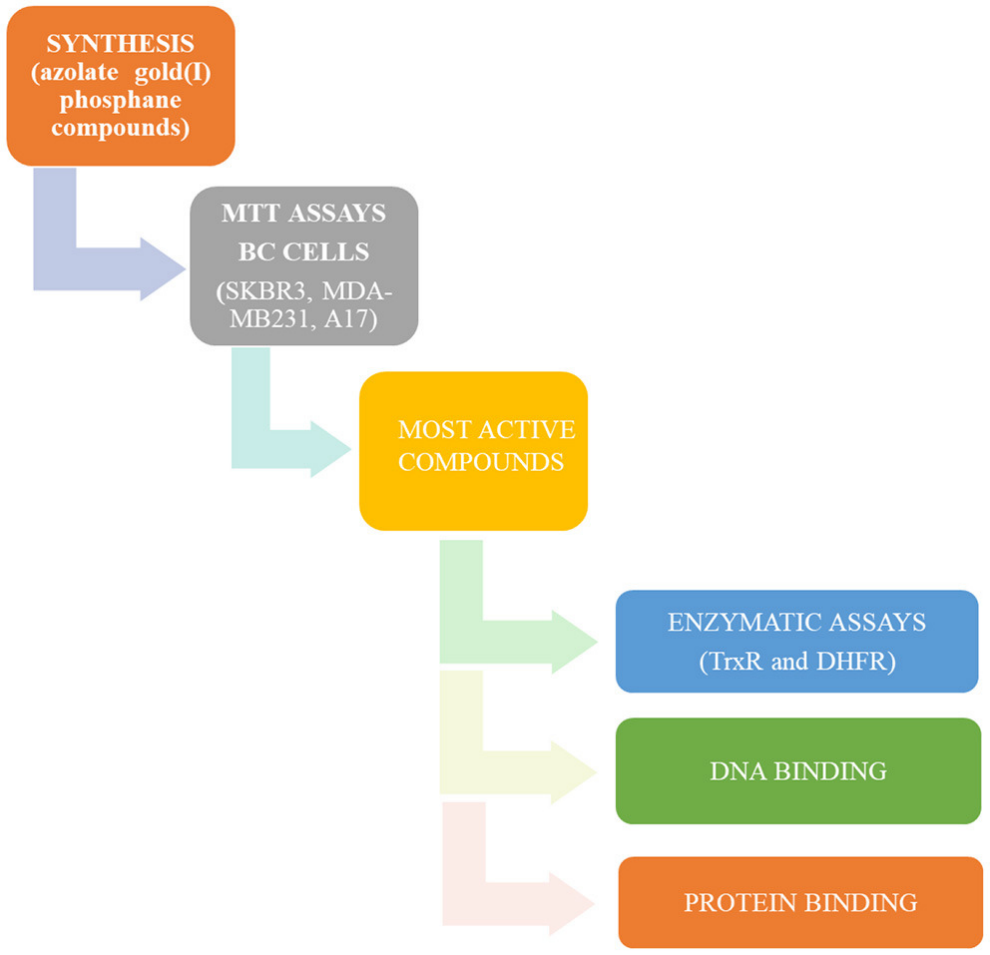
Flow diagram of the experimental plan of this work.

All these compounds were first screened by MTT assay in human SKBR3 cells, representing an HER2-positive BC model. The most effective ones were then tested on both human MDA-MB-231 cells and murine A17 cells, representing BLBC models (Galiè et al., 2005, 2008; Marchini et al., 2010; Bisaro et al., 2012). To shed light on molecular mechanisms underlying the anticancer effects of the selected azole/phosphane gold(I) complexes and to identify new targeted molecules, their inhibitory action on the key cell enzyme dihydrofolate reductase (DHFR) was evaluated using thioredoxin reductase (TrxR) as a reference target. DHFR and TrxR are two enzymes involved in unrelated metabolic pathways, both crucial for cancer cell's growth and survival. They are both overexpressed in tumor cells, and potential molecular target of chemotherapeutic drugs, such as antifolates for DHFR (e.g., methotrexate) (Raimondi et al., 2019), and alkylating agents selectively modifying the selenocysteine (Sec) residue for TrxR (Urig and Becker, 2006). Indeed, TrxR enzyme family has represented an optimal candidate for gold anticancer drugs, based on the strong affinity of the gold atom to thiols, making the nucleophilic selenolate of reduced TrxR the main target site of modification by this metal compounds. Thioredoxin reductase can thus be considered the reference target of gold-based anticancer drugs (Bindoli et al., 2009). Conversely, DHFR does not have selenocysteine residues, and the available thiol groups do belong to cysteines, which are not involved in the catalytic site. Nevertheless, in previous studies we have reported the inhibition of *E. coli* DHFR by gold(I) phosphane complexes with Ki values in the micromolar range (Galassi et al., 2015). Furthermore, supported by thermodynamic analysis, we have ascertained alternative non-covalent mechanisms, by which polyphosphane gold(I) complexes can modify the catalytic behavior of the molecular target without cysteines or selenocysteines modification as in the case of DHFR (Gambini et al., 2018). In this work, we found that phosphane gold(I) complexes can target dihydrofolate reductase by strongly inhibiting its enzymatic activity. In addition, to acquire additional information about the fate of the most active azole/phosphane gold(I) compounds once in contact with serum proteins, targeted spectrophotometric tests were led on BSA (bovine serum albumin) and ATF (apo transferrin): while BSA was already ascertained to bind gold moieties, the ATF, which is a plasmatic protein for iron ions transport, was never taken into account for gold compounds transport. Finally, DNA is not a preferential binding site for gold compounds, hence, to rule out any unusual interaction between some selected gold compounds and calf thymus DNA, spectroscopic studies have been addressed to this last issue. The experimental plan of this work, schematically represented in Figure 1, provides a set of results decisive for an overall assessment of multitarget mechanisms of action for the azolate/phophane gold compounds herein considered, contextually to the confirmation of their strong cytotoxic activity against aggressive BC subtypes.

## Materials and Methods

### Syntheses and Characterization

Elemental analyses (C, H, N, S) were performed in-house with a Fisons Instruments 1,108 CHNS-O Elemental Analyser. Melting points were taken on an SMP3 Stuart Scientific Instrument. IR spectra were recorded from 4,000 to 600 cm^−1^ with a Perkin-Elmer SPECTRUM ONE System FT-IR instrument. IR annotations used: br = broad, m = medium, s = strong, sh = shoulder, vs = very strong, w = weak and vw = very weak. ^1^H and ^31^P NMR spectra were recorded on an Oxford-400 Varian spectrometer (400.4 MHz for ^1^H and 162.1 MHz for ^31^P). Chemical shifts, in ppm, for ^1^H NMR spectra are relative to internal Me_4_Si. ^31^P NMR chemical shifts were referenced to a 85% H_3_PO_4_ standard. The ^31^P NMR spectroscopic data were accumulated with ^1^H decoupling. NMR annotations used: br = broad, d = doublet, dd = double doublet, t = triplet, m = multiplet, s = singlet. Chemicals were purchased from Merck and used without further purification. Electrospray mass spectra (ESI-MS) were obtained in positive- or negative-ion mode on a Series 1,100 MSD detector HP spectrometer, using an acetonitrile or methanol mobile phase. The compounds were added to reagent grade acetonitrile to give solutions of approximate concentration 0.1 mM. These solutions were injected (1 μl) into the spectrometer via a HPLC HP 1090 Series II fitted with an auto-sampler. The pump delivered the solutions to the mass spectrometer source at a flow rate of 300 μl min^−1^, and nitrogen was employed both as a drying and nebulizing gas. Capillary voltages were typically 4,000 and 3,500 V for the positive- and negative-ion mode, respectively. Confirmation of all major species in this ESI-MS study was aided by comparison of the observed and predicted isotope distribution patterns, the latter calculated using the IsoPro 3.0 computer program. The used solvents were HPLC grade and they were used as purchased, unless water and oxygen sensitive reactions were led. Anhydrous and radical free THF was obtained by treating the solvent with Na/acetophenone under N_2_ atmosphere, while other solvents were used as purchased without any additional puification. The azoles, the triphenylphosphane, the 2-diphenylphosphine benzoic acid, and the 4-diphenylphosphine benzoic acid were purchased from Merck and dried under vacuum prior to the use. A foil of metal gold was purchased from Merck and used to synthesize tetrachloridegold(I) acid by dissolving the gold chop by boiling aqua regia and by the careful evaporation of water till almost to dryness. The compound Ph_3_PAuCl was prepared by the reduction of tetrachloridegold(I) acid with a double molar amount of PPh_3_ in ethyl alcohol (Bruce et al., 2007). Compound **1** and compound 4-diphenylphosphine benzoic acid gold(I) chloride were synthesized according to the procedure published in Galassi et al. (2015). The sodium 3,5-dinitropyrazolate was synthesized according to a procedure reported in Galassi et al. (2013). The crystalline samples were characterized according to elemental analysis, IR, melting points, ^1^H and ^31^P NMR spectroscopies, ESI mass spectrometry and the results were compared to previously reported data. The syntheses of compounds **3, 4, 5** and **6** are following reported.

Synthesis of compound **3**, [(4-hydroxy-methyl)imidazolyl-1H-gold(I)-(2-benzoic-diphenylphosphane acid)]


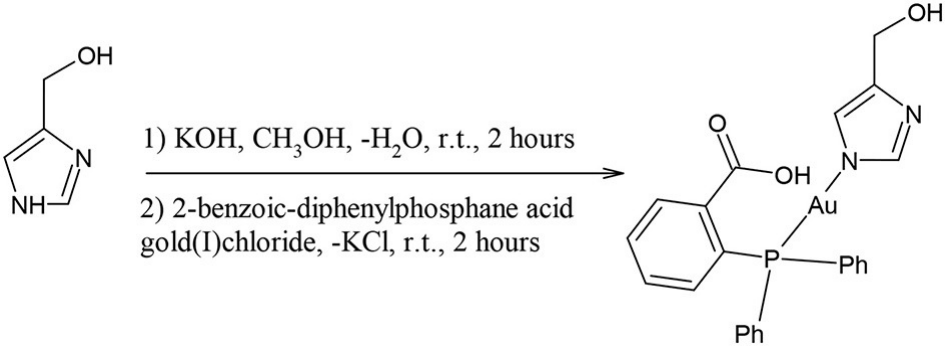
 50 milligrams of solid 4-hydromethyl-imidazole (0.5 mmol) were dissolved in 5 mL of CH_3_OH. To this solution 0.5 mL of a 1 M CH_3_OH solution of KOH (0.5 mmol) were added. After an hour of magnetic stirring at room temperature, 12 mL of a methanol solution containing 275 mgs of compound 1 were added (0.5 mmol). The suspension was stirred for 2 h, concentrated to half volume, and then filtered off over a celite bed. The clear solution was concentrated to 10 mL and let to evaporate till a microcrystalline solid was obtained. Yield 63%. M. p. 195–197°C.

^1^H-NMR (CD_3_OD, δ): 8.11 (m, br 1H); 7.83 (s, 1H), 7.62–7.47 (m, 10H), 7.45 (m, 1H), 7.21 (s, 1H), 7.37 (s, 1H) 6.86 (m, 1H), 4.62 (br, 1H), 4.55 (s, 2H).

^31^P-NMR (CD_3_OD, δ): 31.57 (s).

MIR (cm^−1^): 3,226 (m), 3,123 (m, br), 3,055 (m, br), 2,855 (m, br), 1,631 (m), 1,597 (s), 1,579 (s), 1,562 (sh), 1,557 (s), 1,480 (m), 1,435 (m), 1,371 (s), 1,280 (m), 1,257 (w), 1,241 (m), 1,206 (w), 1,158 (w), 1,101 (s), 1,063 (m), 1,019 (m), 982 (m), 937 (m), 879 (m), 830(m), 747 (s), 711 (s), 692 (vs) FIR (cm^−1^): 654 (m), 619 (m), 549 (m), 512 (m, Au-N) 500 (m), 434 (m), 393 (m), 286 (w, Au-P), 227 (w).

ESI (–) (CH_3_OH, m/z): 493 (100), 537 (50), 599 (20) [(2-COO-Ph_2_P)Au + 4-CH_2_OH-Im]^−^. ESI (+) (CH_3_OH, m/z): 809 (5) [(2-COOH-Ph_2_P)_2_Au]^+^; 600 (100) [(2-COOH-Ph_2_P)Au + 4-CH_2_OH-Im + H]^+^.

Elemental analysis for C_23_H_20_AuN_2_O_3_P, calcd %: C 46.01, H 3.36, N 4.67. Found C 46.36, H 3.71, N 4.99.

Synthesis of compound **4**, [(3,5-dintropyrazolyl-1H-gold(I)-(triphenylphosphane)]


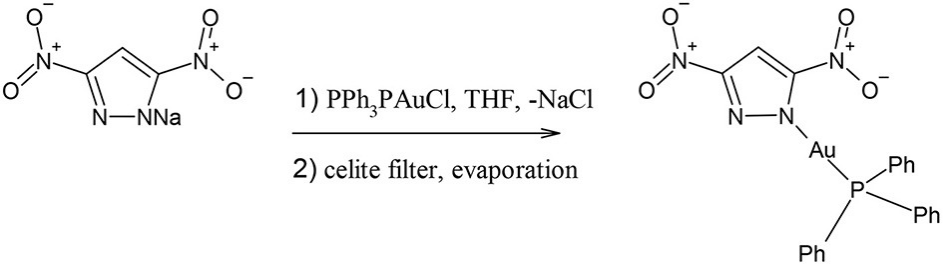
 0.090 g of solid sodium 3,5-dinitropyrazolate (0.5 mmol) were added to 5 mL of THF. To this pale yellow suspension, solid PPh_3_PAuCl was added (0.5 mmol). The suspension was stirred for 3 h, and then filtered off over a celite bed. The celite was washed with 2 mL of THF (3 times) and the clear solution was concentrated to 5 mL. By slow evaporation a microcrystalline solid was obtained. Yield 71%. M. p. 161–163°C.

^1^H-NMR (CD_3_OD, δ): 8.21 (m, br 1H); 7.72–7.41 (m, 15H).

^31^P-NMR (CD_3_OD, δ): 31.20 (s).

MIR (cm^−1^): 3,168 w, 3,054 w, 3,030 w, 1,677 w, 1,585 w, 1,543 m, 1,480 vs, 1,451 s, 1,435 vs, 1,362 vs, 1,326 vs, 1,293 s, 1,181 s, 1,100 vs, 1,066 m, 1,046 m, 1,025 m, 995 m, 923 w, 832 s, 812 s, 740 vs, 711 s, 679 vs.

ESI (+) (CH_3_OH) m/z %: 538.3 (31) [AuPPh_3_]^+^, 721.3 (100) [Au(PPh_3_)_2_]^+^

Elemental analysis for C_23_H_20_AuN_2_O_3_P, calcd %: C 46.01, H 3.36, N 4.67. Found C 46.40, H 3.1, N 4.41.

Synthesis of compound **5**, [(3,5-dicyanoimdazolyl-1H-gold(I)-(triphenylphosphane)]


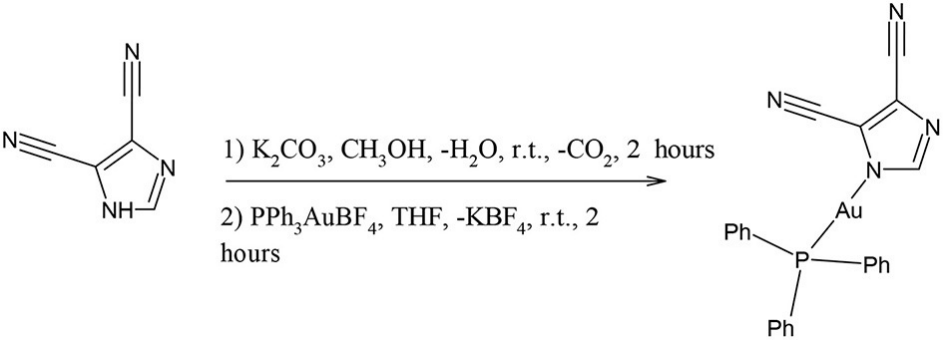
 0.050 g of 4,5-dicyanoimidazole (0.423 mmol) was dissolved in 4 mL of methanol. To this solution, K_2_CO_3_ (0.059 g; 0.423 mmol) dissolved in few drops of water was added. The colorless solution was stirred overnight and added to a 4 mL of freshly prepared Ph_3_PAuBF_4_ in THF solution (0.423 mmol, the THF solution of Ph_3_PAuBF_4_ is prepared by adding a 1:1 mole ratio ammount of AgBF4 and by filtering through a celite bed). The cloudy solution was stirred for 3 h, filtered and the solution was evaporated to dryness. A white solid was obtained and crystallized by a mixture of CH_2_Cl_2_- diethyl ether. Yield 59%.

^1^H NMR (CD_3_OD, δ): 7.80 (s, 1H), 7.67÷7.59 (m, 15H).

^31^P NMR (CD_3_OD, δ): 30.63(s).

IR (cm^−1^): 3214.4 (br, w), 3,047 (w), 2,228 (m), 1,572 (br, m), 1,499 (m), 1,471 (m), 1480.5 (m), 1,446 (sh, m), 1,435 (vs), 1331.5 (m), 1,303 (m), 1,249 (m),1,202 (m), 1,181 (m), 1159.3 (w), 115 (m), 1,103 (s), 1,072 (m), 1,027 (m), 998 (m), 920 (m), 878 (s), 849 (w), 754 (m), 745 (s), 712 (s), 692 (vs), 661 (vs).

ESI(–) (CH_3_OH) m/z, %: 117 (71) [4,5-ImCN_2_]^−^, 431 (100) [(4,5-ImCN_2_)_2_ + Au]^−^.

ESI(+) (CH_3_OH) m/z %: 721 (100) [(PPh_3_)_2_Au]^+^, 1,035 (16).

Elemental analysis for C_23_H_16_N_4_PAu calcd %: C 45.69, H 2.86, N 9.60. Found %: C 46.02; H 2.66, N 9.23.

Synthesis of compound **6**, [(3,5-dichloroimdazolyl-1H-gold(I)-(triphenylphosphane)]


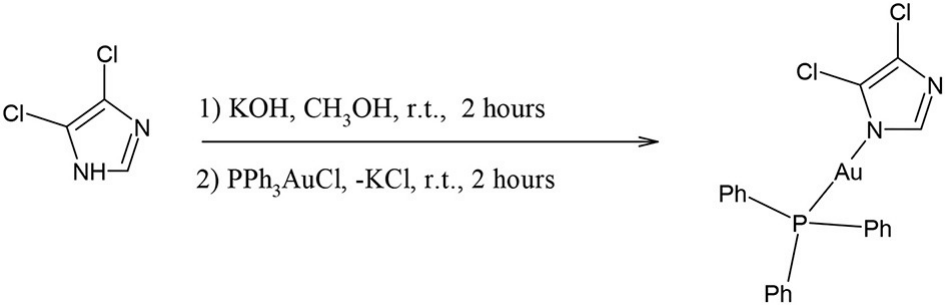
 0.1 g of solid 4,5-dichloroimidazole (0.168 mmol) were dissolved in 2 mL of CH_3_OH. To this solution 1.7 mL of a 0.1 M CH_3_OH solution of KOH (0.168 mmol) were added. After 2 h of magnetic stirring at room temperature, 83 mgs of solid Ph_3_PAuCl was added (0.168 mmol). The suspension was stirred for further 2 h, filtered off over a celite bed. The clear solution was concentrated to dryness, dissolved in CH_2_Cl_2_ and crystallized by layering hexane. Yield 59%. M.p. 154÷156.5°C.

^1^H NMR (CD_3_OD, δ): 7.74÷7.64 (m, 15H), 7.20 (s, 1H),

^31^P NMR (CD_3_OD, δ): 31.48 (s).

IR (cm^−1^): 3,150 w (C_2_-Him), 3,054 w, 3,032 w, 1,587 m, 1,499 m, 1,480 m, 1,434 s, 1,331 m, 1,309 m, 1,259 m, 1,222 s, 1,188 m, 1,101 s, 1,041 m, 1,011 s, 997 m, 963 m, 920 m, 812 m, 743 s, 711 s, 689 vs.

ESI(+) (CH_3_OH), m/z (%): 1053.1 (100) [Au(PPh_3_)_2_ + 4,5-Cl_2_Im]^+^.

ESI(–) (CH_3_OH), m/z (%): 134.9 (100) [4,5-Cl_2_Im]^−^, 468.9 (40) [Au(4,5-Cl-Im)_2_]^−^.

Elemental analysis for C_21_H_16_AuCl_2_N_2_P, calcd %: C, 42.38, H, 2.71, N, 4.71. Found %: C, 42.02, H, 2.84, N, 4.52.

### Cancer Cells

Human MDA-MB-231 (ER–/PR–/HER2–) and SK-BR-3 (ER–/PR–/HER2+) cells were obtained from American Type Culture Collection (Rockville, MD) and cultured in Dulbecco's Modified Essential Medium (DMEM, Gibco, Life Technologies) supplemented with 10% fetal bovine serum (FBS, Gibco, Life Technologies) and 1% penicillin–streptomycin (Gibco, Life Technologies). A17 cells were established from a spontaneous lobular carcinoma that arose in an FVB/neuT mouse (Galiè et al., 2005), and maintained in DMEM plus 20% FBS and 1% penicillin-streptomycin. Cells were cultured at 37°C under humidified atmosphere with 5% CO_2_.

### MTT Assays

Stock solutions were prepared for each gold(I) compound (50 mM) using dimethyl sulfoxide (DMSO) (Sigma-Aldrich) as solvent. The effects of gold(I) compounds on SKBR3, A17 and MDA-MB231 cells viability were evaluated by seeding, respectively, 10, ^*^10^3^, 2.5 ^*^10^3^, 7, ^*^10^3^ cells/well in 96 wells plates in complete medium (DMEM supplemented with FBS) and compared to those of cisplatin. The day after, fresh medium containing vehicle alone (DMSO, 1 % v/v final concentration) or increasing concentrations of each compound ranging from 0.1 to 100 μM was added. Cell viability was determined using a MTT [3-(4,5-dimethylthiazol-2-yl)-2,5-diphenyl-2H-tetrazolium bromide Sigma Aldrich, St. Louis, MO] assay, which is based on the conversion of MTT to formazan crystals by mitochondrial enzymes, after 24, 48, and 72 h. The formazan deposits were dissolved in DMSO and the absorbance of each well was measured at 540 nm in Multiskan Ascent 96/384 Plate Reader. Each drug concentration was evaluated with 8 replicates and each experiment was repeated three times. At the end of the experiments a dose–response curve was plotted, and the IC50 concentrations were determined. Statistical analysis was performed using One-Way ANOVA analysis of variance followed by Dunnett's Multiple Comparison Test. Quantitative data are presented as means ± SEM. Differences were considered significant at ^*^P ≤ 0.0332; ^**^P ≤ 0.0021; ^***^P ≤ 0.0002; ^****^P ≤ 0.0001.

### Enzymatic Assays on DHFR

SKBR3, MDA-MB-231 and A17 cells were plated onto 100 mm cell culture dishes (2 × 10^6^ cells/ dish). The day after, cells were treated with vehicle alone (DMSO) as control or increasing concentrations of compound **5** and compound **6** in DMEM supplemented with 2% FBS (Invitrogen, Carlsbad, CA) for 4 h or 12 h. Representative pictures of cells treated with compound **5** or **6** at the indicated concentration for 4 or 12 h just before to take cell lysates for the enzymatic tests are reported as supplemental files. Cell lysates were collected using Cell Culture Lysis Reagent (Promega, Madison, WI) supplemented with BSA (1%) and protease inhibitors. DHFR activity was assessed by spectrophotometric assay as reported elsewhere (Wang et al., 2020) in which the reaction rate was followed by recording the decreasing absorbance at 340 nm due to oxidation of NADPH to NADP^+^, using a Shimadzu UV-2450 spectrophotometer. When necessary, because of low protein content in the lysates, the DHFR enzymatic activity was measured by a discontinuous direct HPLC assay using an Agilent 1100 system. HPLC-DAD (Agilent 1100) was used to detect and quantify the presence of NADP^+^, as one of the products of DHFR catalyzed reaction, in cell lysates. Fifty microliter of the cell lysate were incubated at 37°C in a reaction mixture containing 760 μl of buffer (0.05 M Hepes pH 7.3), 20 μl of NADPH 4 mM, and 20 μl of dihydrofolate (H_2_F) 4 mM. The samples for HPLC analysis were prepared as follows: 50 μl aliquots of each mixture were stopped at different times (0, 5, 10, 20, 30, and 60 min) by adding 25 μl of HClO_4_ 1.2 M; after 10 min on ice and centrifugation for 5 min at 12,000 g, 60 μl of each supernatant were neutralized by adding 17.5 μl of K_2_CO_3_ 0.79 M, kept on ice and centrifuged again. Fifty microliter of the supernatant obtained were directly injected into the reversed phase column for separations of reagents and products of the enzyme catalyzed reaction. The column used is a Supelcosil LC RP 18 (4.6 × 250 mm) with pre-column SupelGuard. The HPLC settings are the same reported in Wang et al. (2020). The amounts of product (NADP^+^) were determined from the peak areas of the HPLC-separated compounds with reference to appropriate standards. One enzyme unit is defined as the amount of enzyme which catalyzes the reduction of 1 μmol of dihydrofolate, per minute, at 37°C. Enzyme activities were normalized by the protein content, determined by the Bradford assay (Bradford, 1976).

### Enzymatic Assays on TrxR

The assay was performed as previously reported (Galassi et al., 2012) in 0.2 M Na, K-phosphate buffer (pH 7.4) containing 2 mM EDTA. The assay contained 0.12 mM NADPH and different amounts of cell lysate. The reaction was initiated by the addition of 3 mM 5,5′-dithiobis (2-nitrobenzoic acid) (DTNB) and the increase of absorbance was monitored at 412 nm over 5 min at 25°C.

### DNA Interaction Studies

Stock solutions of calf thymus DNA (ct-DNA) (Sigma–Aldrich) were prepared by dissolving the DNA powder overnight in 10 mM Tris–HCl pH 7.4, 10 mM NaCl and incubated under stirrer at 4°C for 12h. The UV absorbance at 260 and 280 nm of the DNA solution gave a ratio A_260_/A_280_ solution of ct-DNA of ca. 1.9, indicating that the DNA was sufficiently free from protein (Xu et al., 2014). The concentration of the ct-DNA solution was determined by UV absorbance at 260 nm. The molar absorption coefficient at 260 nm, was taken as 6,600 M^−1^ cm^−1^ (Marmur, 1961). Stock solutions of gold(I) complexes (compounds **5** and **6**) were prepared in DMSO. Absorption titration experiments performed with fixed concentrations of the different gold(I) complexes (25 μM, in 10 mM Tris–HCl pH 7.4, 10 mM NaCl), gradually increasing the concentration of DNA. After addition of DNA to the compounds, the resulting solution was allowed to where equilibrate at room temperature for 5 min. Then, the sample solution was scanned in the range 220–600 nm. For the different compounds the binding constant (Kb) was determined from the spectroscopic titration data using the following equation

$$\begin{array}{l} \left[\text{DNA}\right]/\left(\varepsilon_{\text{a}}-\varepsilon_{\text{f}}\right)=\left[\text{DNA}\right]/\left(\varepsilon_{\text{b}}-\varepsilon_{\text{f}}\right)+1/\text{Kb}\left(\varepsilon_{\text{b}}-\varepsilon_{\text{f}}\right),\text{Equation }1 \end{array}$$

where [DNA] is the concentration of DNA in base pairs, the apparent absorption coefficient (ε_a_) was obtained by calculating A_obsd_/[compound]. The terms ε_f_ and ε_b_ correspond to the extinction coefficient of free (unbound) and the fully bound compound, respectively. A plot of [DNA]/(ε_a_-ε_f_) vs. [DNA] will give a slope 1/(ε_b_-ε_f_) and an intercept 1/Kb(ε_b_-ε_f_). Kb is given by the ratio of the slope to the intercept.

#### Fluorescence Spectral Study With EtBr

EtBr weakly emits fluorescence in aqueous solutions (Pasternack et al., 1983), however in presence of ct-DNA strongly emits at ~600 nm due to strong intercalation between adjacent DNA base pairs. Ethidium bromide (EB) can be employed as a probe for the spectroscopic study of the interaction between DNA and intercalating species because of a 24-fold decrease in its fluorescence when displaced from an intercalation site by the addition of a competitive binding molecule probably due to decreasing in the number of binding sites accessible to EtBr (Tong et al., 2012). Competitive binding experiments were performed maintaining the ethidium bromide (EB) and ct-DNA concentration at 5 μM and 55.7 μM, respectively, while increasing concentrations of compounds **5** and **6** (5 mM in DMSO) were added to the buffer solution (10 mM Tris–HCl pH 7.4, 10 mM NaCl). Fluorescence quenching spectra were recorded using a Hitachi 4,500 spectrofluorometer with an excitation wavelength of 490 nm and emission spectrum 500–700 nm. The fluorescence spectra were recorded and the fluorescence value of the decrease in emission spectra was corrected according to the relationship:

(1) $$\begin{array}{l} {\text{F}}_{\text{c}}={\text{F}}_{\text{m}}\times \text{e}\left({\text{A}}_{1}+{\text{A}}_{2}\right)/2 \end{array}$$

where Fc and Fm are the corrected and measured fluorescence, respectively. A1 and A2 are the absorbance of tested compounds at the exciting and emission wavelengths. For fluorescence quenching experiments, Stern-Volmer's equation was used (Equation 3):

(2) $$\begin{array}{l} {\text{F}}_{0}/{\text{F}}_{c}=1+\text{kq}\tau 0\left[\text{C}\right]=1+{\text{K}}_{\text{sv}}\left[\text{C}\right] \end{array}$$

where F_0_ and F_c_ represent the fluorescence intensity in the absence and in the presence of the metal complex, [C] is the concentration of the metal complex and Ksv is the Stern-Volmer constant that can be obtained from fluorescence data plotted as F0/Fc vs. the metal complex concentration [C] (Lakowicz, 2006). All experiments involving ct-DNA were performed in buffer solution (10 mM tris-HCl buffer pH 7.4 10 mM NaCl) at room temperature.

#### Minor Groove Displacement Assay

The changes in the emission spectra of DAPI complex with the DNA in 10 mM tris-HCl buffer at pH 7.4, 10 mM NaCl, were monitored upon addition of increasing concentrations of gold-compounds **5** and **6** at room temperature. Fluorescence quenching data were evaluated after excitation of DAPI-ct-DNA complex at 338 nm and recording the spectra from 400 to 600 nm. Values of Ksv constants were evaluated using Equation 3 and the quenching fluorescence values obtained were corrected according to Equation 2.

### Protein Binding

• BSA binding

The protein-binding studies were performed by tryptophan fluorescence quenching experiments using bovine serum albumin (BSA) from Sigma Aldrich prepared in 10 mM Tris-HCl buffer pH 7.4, 10 mM NaCl. The concentration was determined by measuring the absorbance at 280 nm. Assuming a molecular weight of 66,400 and a molar extinction coefficient at 280 nm of 43,824 M^−1^ cm^−1^. Fluorescence measurements were recorded on a Hitachi 4,500 spectrofluorometer by keeping the concentration of BSA constant (15 × 10^−6^ M) while increasing compounds **5** or **6** concentrations (5 mM in DMSO) were added to protein solution at room temperatures. Protein fluorescence intensity was recorded after each successive addition of gold compound solution and equilibration (ca. 5 min). Fluorescence spectra were recorded from 300 to 450 nm at an excitation wavelength of 285 nm. The value of Stern Volmer constants of the different metal complexes to BSA was evaluated following Equation 3 and fluorescent values were corrected by Equation 2.

• Transferrin binding

Human apoTF (ATF) was purchased from Sigma Aldrich and its concentration was determined by measuring the absorbance at 278 nm (ε = 92,300 M^−1^ cm^−1^). To evaluate if compounds **5** and **6** were able to bind to ATF *in vitro*, fluorescence spectra were recorded from 300 nm to 450 nm upon tryptophan excitation at 285 nm. Fluorimetric titrations were performed by titrating a 2 mL sample (prepared in 10 mM Tris-HCl buffer pH 7.4, 10 mM NaCl) containing human ATF (12.5 μM) with incremental additions of different aliquots of gold-complexes (5 mM in DMSO). Protein fluorescence intensity was recorded after each successive addition of complex solution and equilibration (5 min). The value of Stern Volmer constants of the different metal complexes to ATF were evaluated following Equation 3 and fluorescent values were corrected by Equation 2. All titrations were performed at room temperature (25°C) and averages between two trials are reported.

## Results

### Syntheses

The gold(I) compounds tested in this work are shown in Scheme 1 and listed as compounds **1**-**6**. The nature of their molecular structure was ascertained by comparing the characterization results with the data previously reported in literature (*vide infra*). They were prepared as microcrystalline powders and used in the biochemical assays upon dissolution in DMSO. As concern compound **3**, the structure was mostly attributed from the elemental analysis, the ESI-MS and IR data. In detail, the IR spectrum displays a large band attributable to the OH group centered at 3226 cm^−1^ and a band at 1631 cm^−1^ which appears 52 cm^−1^ redshifted from the 1683 cm^−1^ carbonyl band of the starting 2-COOH-Ph_2_PAuCl; moreover, the overall ^1^H NMR patterns and the ESI(–) and ESI(+) MS peaks at 599 and 600 m/z, attributed to [M]^−^ and [M + H]^+^ ions, respectively, are diagnostic for the molecular structure of compound **3** reported in Scheme 1.

### MTT Tests on Breast Cancer Cells

**Figure 2:**
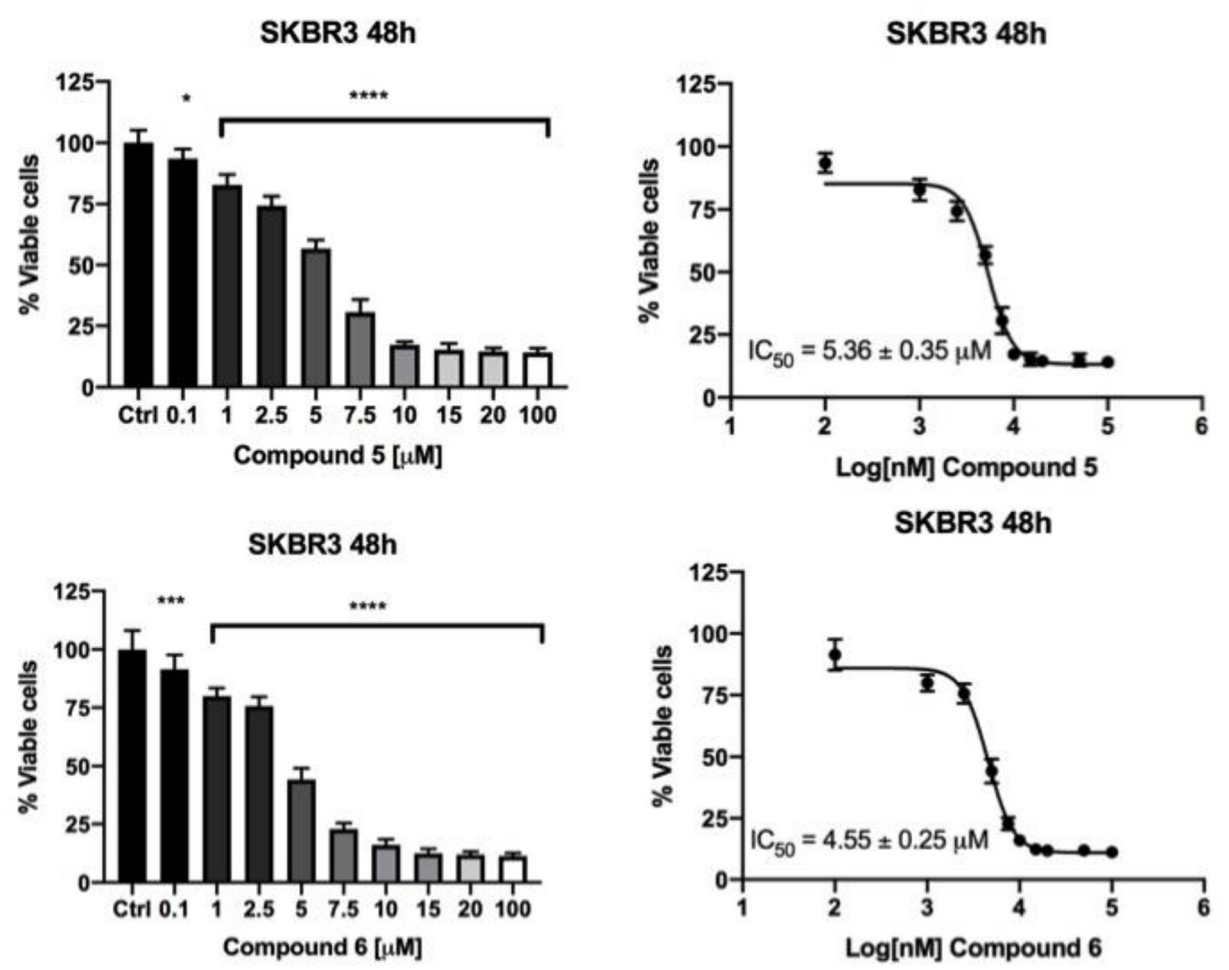
Compounds **5** and **6** decreased SKBR3 cell viability. SKBR3 cells were incubated for 48 h in the presence of vehicle (DMSO) or increasing concentrations of Compound 5 (upper panel) and Compound 6 (lower panel) and cell viability was determined by MTT assay. The results are expressed as percentage of living cells with respect to control (vehicle alone). Columns, mean of three separate experiments wherein each treatment was repeated in 8 wells; bars, SE. *P ≤ 0.0332; ***P ≤ 0.0002; ****P ≤ 0.0001, One-way ANOVA followed by Dunnett's multiple comparisons test. IC_50_ values were calculated, by fitting the concentration-effect curves data obtained in the three experiments with the sigmoid-E_max_ model using non-linear regression, weighted by the reciprocal of the square of the predicted effect.

The effect on cancer cell viability of the gold(I) compounds reported in Scheme 1 was first estimated by MTT assay on SKBR3 cells, representing the HER2-positive breast cancer subtype, after drug treatment for 72 h (Figure 1S). Considering the greater efficacy displayed by compounds **5** and **6** with respect to the others, we selected them for further studies. As shown in Figure 2 and in Table 2, the viability of SKBR3 cells, exposed to compounds **5** and **6** for 48 h, significantly decreased in a dose-dependent manner, with IC_50_ values at low μM concentrations. The IC_50_ values were compared to those of cisplatin, a currently used chemotherapeutic drug (Serova et al., 2006; Gambini et al., 2018). The tests were performed also for the free azolate ligands, as controls, revealing absent or negligible cytotoxic activities (data not shown). Then, this study was extended to BLBC models. For this purpose, human MDA-MB231and murine A17 cells were treated with compounds **5** and **6** for 48 h and their viability was evaluated by MTT assay (Figure 2S). As reported in Table 2, IC_50_ values for compounds **5** and **6** indicate that 48 h of treatments with low μM concentrations are enough to significantly decrease A17 and MDA-MB231 cell viability (see also Figure 2S). These data confirm the previously obtained results in BLBC models, reported by Gambini et al. (2018).

**Table 2:** IC_50_ values for compounds 4, 5, 6 and cisplatin against the BC cell lines SKBR3, MDAMB231, and A17.

**Gold(I) compound**	**Cell line**	Time (h)^*^	**IC_**50**_ [μM ± S. D.]**
Compound **4**	SKBR3	24	12.28 ± 1.1
	MDA-MB231	24	12.85 ± 0.7
	A17	24	11.00 ± 1.0
Compound **5**	SKBR3	48	5.36 ± 0.35
	MDA-MB231	48	7.03 ± 0.76
	A17	48	16.19 ± 0.59
Compound **6**	SKBR3	48	4.55 ± 0.25
	MDA-MB231	48	3.46 ± 0.29
	A17	48	14.43 ± 0.7
Cisplatin	SKBR3	24	43 ± 7.0^∧^
	MDA-MB231	48	50.49 ± 2.0^*^
	A17	24	15.86 ± 1.17^*^

∧ Serova et al., 2006;

* *Gambini et al., 2018*.

Finally, the gold(I) compound having 3,5(NO_2_)_2_-pyrazolate (compound **4**), known to be cytotoxic (Galassi et al., 2012), was introduced in this analysis to evaluate the role of the azole in the anticancer activity. Thus, SKBR3, MDA-MB231 and A17 cells were treated with compound **4** for 24 h and their viability was assessed. The obtained data (Table 2) demonstrate that, besides compounds **5** and **6**, also the gold compound **4** is effective against both HER2-positive and basal like BC models.

### Enzymatic Studies on DHFR

**Figure 3:**
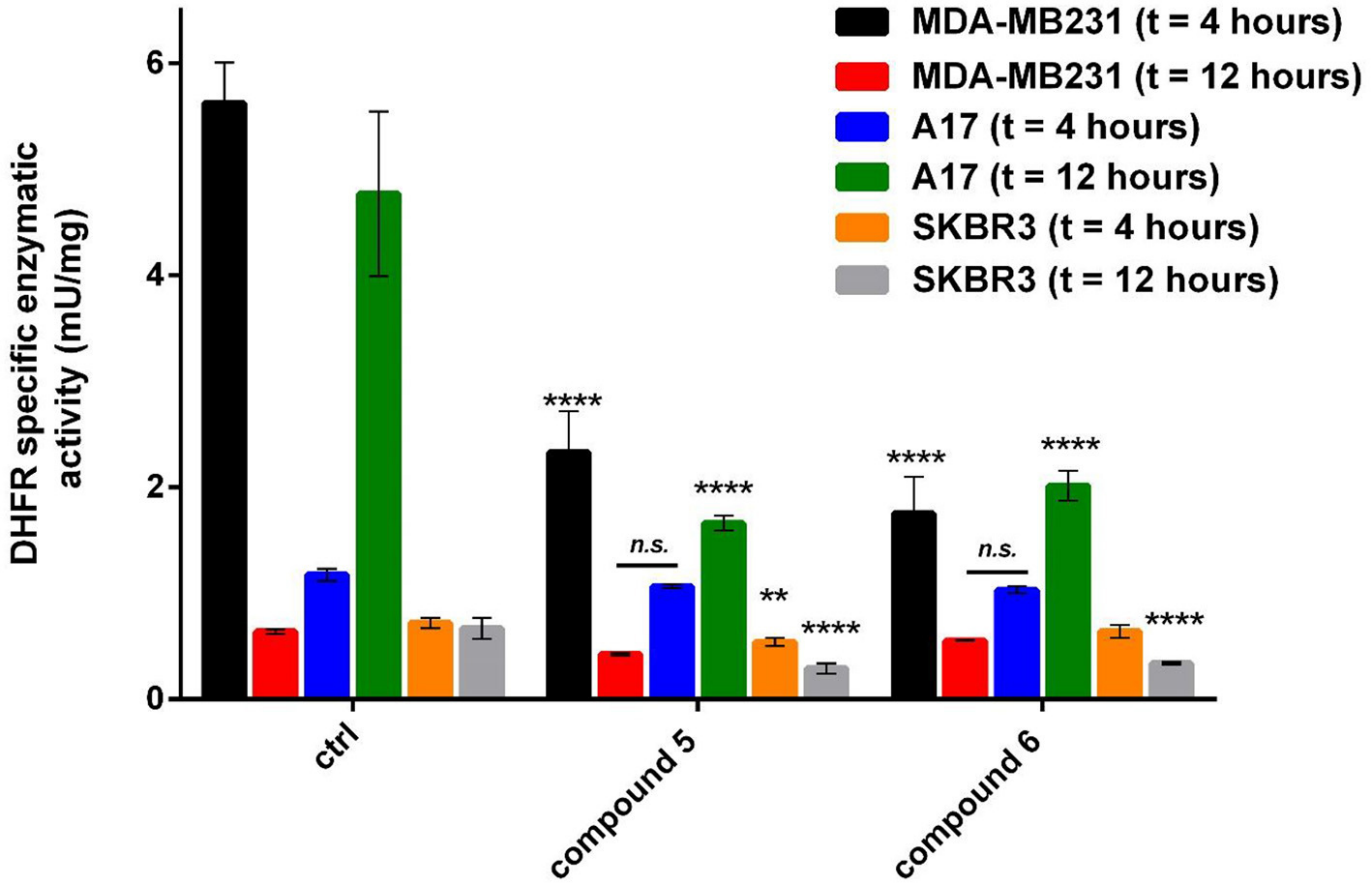
Specific DHFR enzymatic activity measured in breast cancer cell lines after treatment with gold compounds **5**, 4,5-dicyano-imidazolate-1yl-gold(I)-(triphenylphosphane), and **6**, 4,5-dichloro-imidazolate-1yl-gold(I)-(triphenylphosphane), at two different times of treatment. The reported enzymatic activity are relative to the concentration of gold(I) compounds causing the maximum inhibition: 6 μM for A17 and SKBR3, 8 μM for MDA-MB231. The statistical significance relative to control for each cell line is reported: ****p< *0.0001*, **p< *0.01, n.s. not significant*.

The effects of the most active compounds **5** and **6** on the DHFR enzymatic activity were investigated. The inhibition activity on the dihydrofolate reductase (DHFR) has been measured in BC cells treated with gold complexes **5** and **6** within either 4 or 12 h upon treatment and in not treated cells, at the same conditions, as control. Several concentrations of compound **5** and **6** were tested, based on the results of IC_50_ values obtained by MTT assays. In Table 3 the DHFR specific enzymatic activity values, normalized with respect to the total protein content measured in each cell lysate, are shown. Both compounds **5** and **6** have produced a strong inhibition of DHFR enzymatic activity in all the tumor cell lines, reducing in some cases, the enzymatic activity to 35–40% of the control. In all the cancer cells, it was possible to observe a different effect depending on the time of treatment, as clearly evidenced in Figure 3. Taking into account the time necessary for the drugs to enter into the cells and interfere with the enzyme expression and functional activity, the A17 and SKBR3 cells are more efficiently inhibited at 12 h of treatment by both compounds **5** and **6**, while in the case of MDA-MB231 the stronger effect is observed at 4 h of treatment. Remarkably, the specific activity of DHFR in the untreated MDA-MB231 cells at 4 h is higher than the one measured at 12 h. This difference in the DHFR enzymatic levels between the two time points reflects the different inhibitory effects of compounds **5** and **6** on the DHFR activity.

**Table 3:** Specific enzymatic activity of DHFR (mU/mg) measured in the untreated cells and upon treatment with the selected gold(I) compounds at the concentration causing the maximum inhibition: 6 μM for both A17 and SKBR3, 8 μM for MDA-MB231.

	**Time (hours)**	**A17**	**MDA-MB231**	**SKBR3**
Cells without treatment (control)	4	1.17 ± 0.06	5.38 ± 0.38	0.72 ± 0.05
	12	4.77 ± 0.78	0.64 ± 0.02	0.67 ± 0.10
Cells treated with compound **5**	4	1.07 ± 0.02	2.37 ±0.39	0.54 ± 0.04
	12	1.66 ± 0.07	0.42 ± 0.01	0.29 ± 0.05
Cells treated with compound **6**	4	1.03 ± 0.03	1.75 ± 0.34	0.64 ± 0.06
	12	2.01 ± 0.14	0.55 ± 0.002	0.34 ± 0.01

### Enzymatic Studies on TrxR

**Figure 4:**
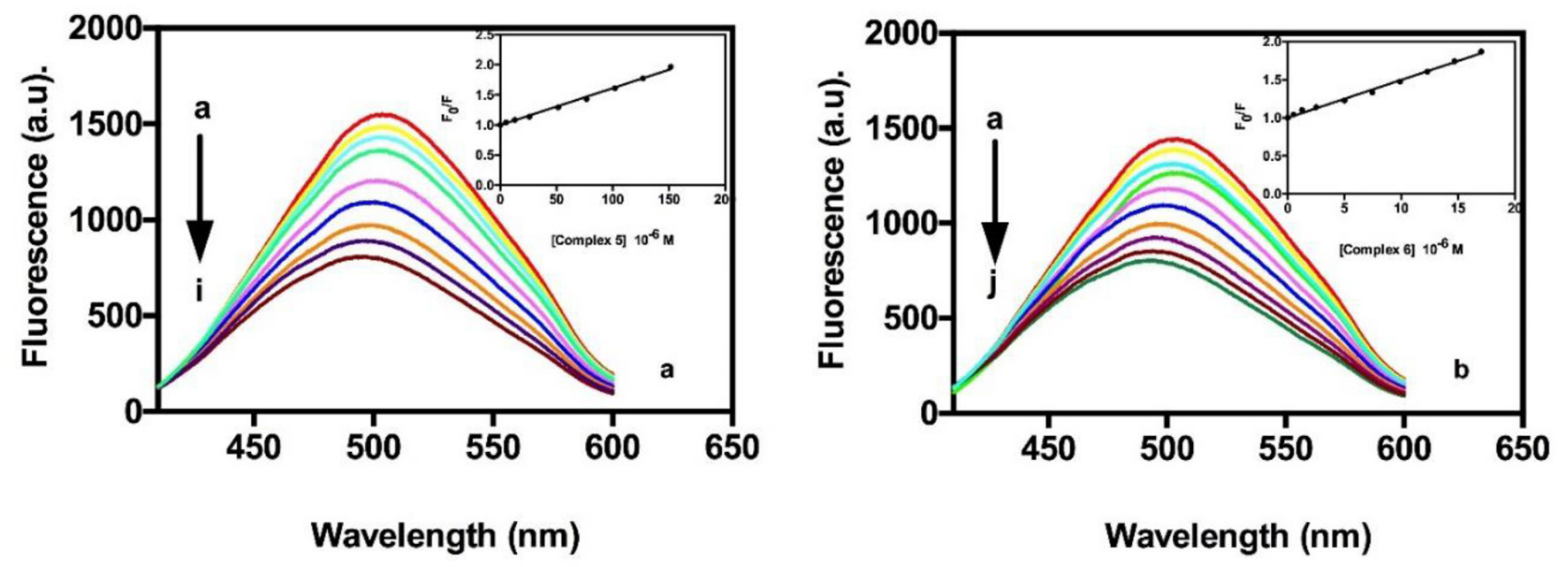
Quenching behavior in fluorescence of the DAPI-ctDNA in the presence of different amounts of compound **5** (a) and compound **6** (b). The arrow indicates the changes of the bands upon addition of increasing concentrations of compounds **5** or **6**. Inset, Stern-Volmer plot of experimental data fitted by Equation 3.

Complexes **5** and **6** were tested for their skill to inhibit the mostly recognized molecular target for gold compounds, the TrxR. With this aim, we evaluated the specific activity of TrxR on different tumor cells SKBR3, A17 and MDA-MB231 after incubation with the gold complexes **5** and **6**. The specific activity data in mU/mg are reported in Table 4. Residual activity data were reported in Figures 3S, 4S where we can observe that thioredoxin reductase activity decreases in a dose dependent manner upon treatment with the two gold(I) compounds with a major effect on MDA-MB231 with respect to those reported for A17.

**Table 4:** Specific enzymatic activity of TrxR mU/mg measured in the untreated cells and upon treatment of 12 h with the selected gold(I) compounds at the concentration causing the maximum inhibition (in round brackets).

	**A17**	**MDA-MB231**	**SKBR3**
Cells without treatment (control)	4.95 ± 0.08	3.01 ± 0.01	4.07 ± 0.32
Cells treated with compound **5**	1.84 ± 0.06 (6 μM)	1.59 ± 0.03 (16 μM)	1.98 ± 0.06 (8 μM)
Cells treated with compound **6**	4.21 ± 0.09 (6 μM)	1.95 ± 0.06 (16 μM)	1.51 ± 0.04 (8 μM)

### Interactions With ct-DNA

#### UV-Vis Absorption Titration Analysis

**Figure 5:**
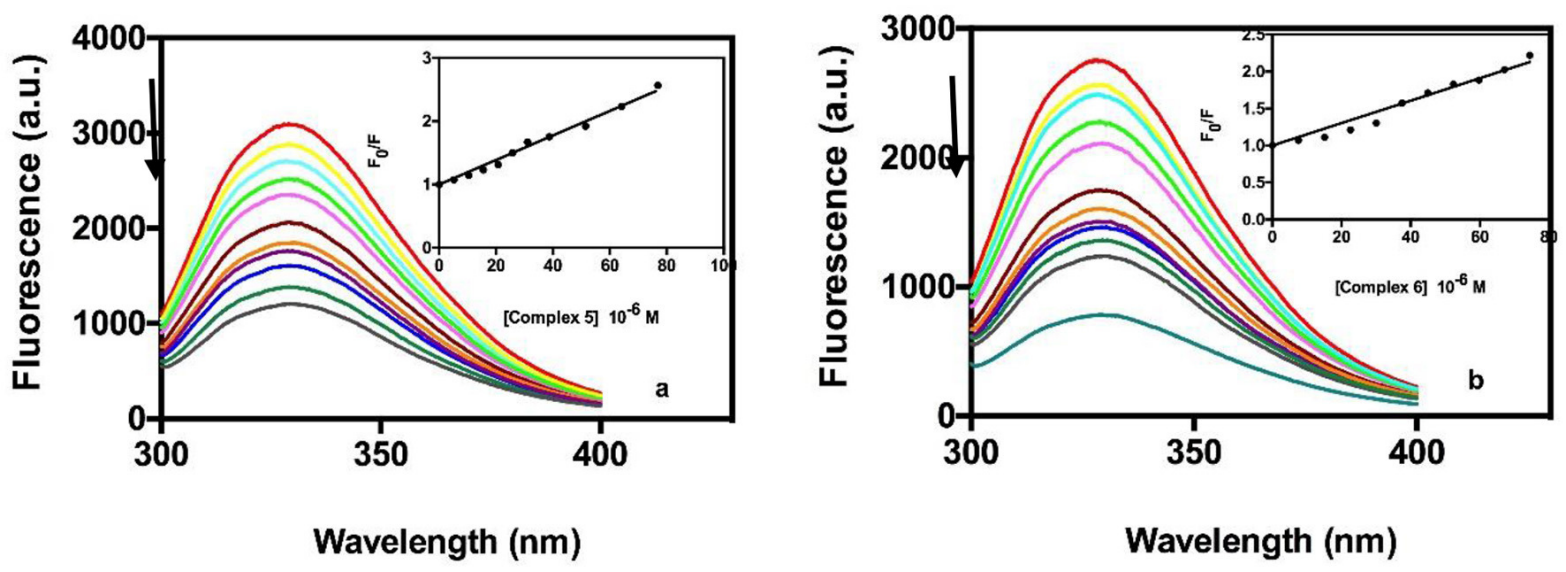
Quenching behavior in fluorescence of the ATF in the presence of different amounts of compound 5 (a) and compound 6 (b). The arrows indicate the changes of the bands upon addition of increasing concentrations of compounds 5 or 6. Inset, Stern- Volmer plot of experimental data fitted by Equation 3.

Bindings between small molecules and DNA represent one of the primary mechanism of cytotoxic activity for metal based drugs. Experiments based on UV-visible spectrophotometric titrations were led to obtain dissociation constants (Kb) in order to compare the binding properties of the selected cytotoxic gold(I) complexes with DNA. The absorption spectra of compound **5** and compound **6** were recorded in the absence and in the presence of calf thymus DNA (ct-DNA) (reported in Figure 5S) while the Kb values for gold compounds **5** and **6** are listed in Table 5. The titration was performed by using fixed amounts of complexes (25μ mM in DMSO**)** and titrating with an increasing amount of ct-DNA, resulting in an overall hyperchromism at the intraligand absorption bands in the 240–300 nm range which is attributed to π-π^*^ transitions. The “hyperchromic effect” has been observed for both tested compounds, in addition to a moderate red shift of the maxima of about 5 nm. The evaluation of the binding affinity of complexes with ct-DNA, was determined by the calculation of the intrinsic binding constants Kb by using the Equation 1, from the plot [DNA]/[ε_a_-ε_f_] vs. [DNA] (Figure 3S) and values calculated for all complexes were reported in Table 5. The Kb analysis confirms that the two complexes interact with DNA with low affinity (compound **5** Kb = 1.46 ± 0.12 10^3^ M^−1^; compound **6** Kb = 3.44 ± 0.37 10^3^ M^−1^) reinforcing the concept of different mechanisms for their cellular toxicity. Among the two compounds, compound **6** shows a non-significant increase on interaction with DNA having 2-folds the value of Kb if compared to compound **5**.

**Table 5:** Summarizing table showing K_sv_ obtained from fluorescence quenching experiments to evaluate compound 5 and compound 6 capacity to bind to proteins (BSA and ATF) and ct-DNA (Competitive binding fluorescence studies with DAPI and EB).

**Compounds**	**BSA** **Ksv 10^**4**^ M^**−1**^**	**ATF** **Ksv 10^**4**^ M^**−1**^**	**Ct-DNA** **Kb 10^**3**^ M^**−1**^**	**EB-ct-DNA** **Ksv 10^**3**^ M^**−1**^**	**DAPI-ct-DNA** **Ksv 10^**3**^ M^**−1**^**
**5**	2.46 ± 0.82	1.93 ± 0.44	1.46 ± 0.12	1.55 ± 0.34	6.12 ± 0.09
**6**	2.04 ± 0.71	1.51 ± 0.55	3.44 ± 0.37	4.22 ± 0.34	4.98 ± 0.08

*K_b_ were obtained through UV-Vis absorption titration analysis*.

#### Competitive Binding Fluorescence Studies With EtBr

**Figure 6:**
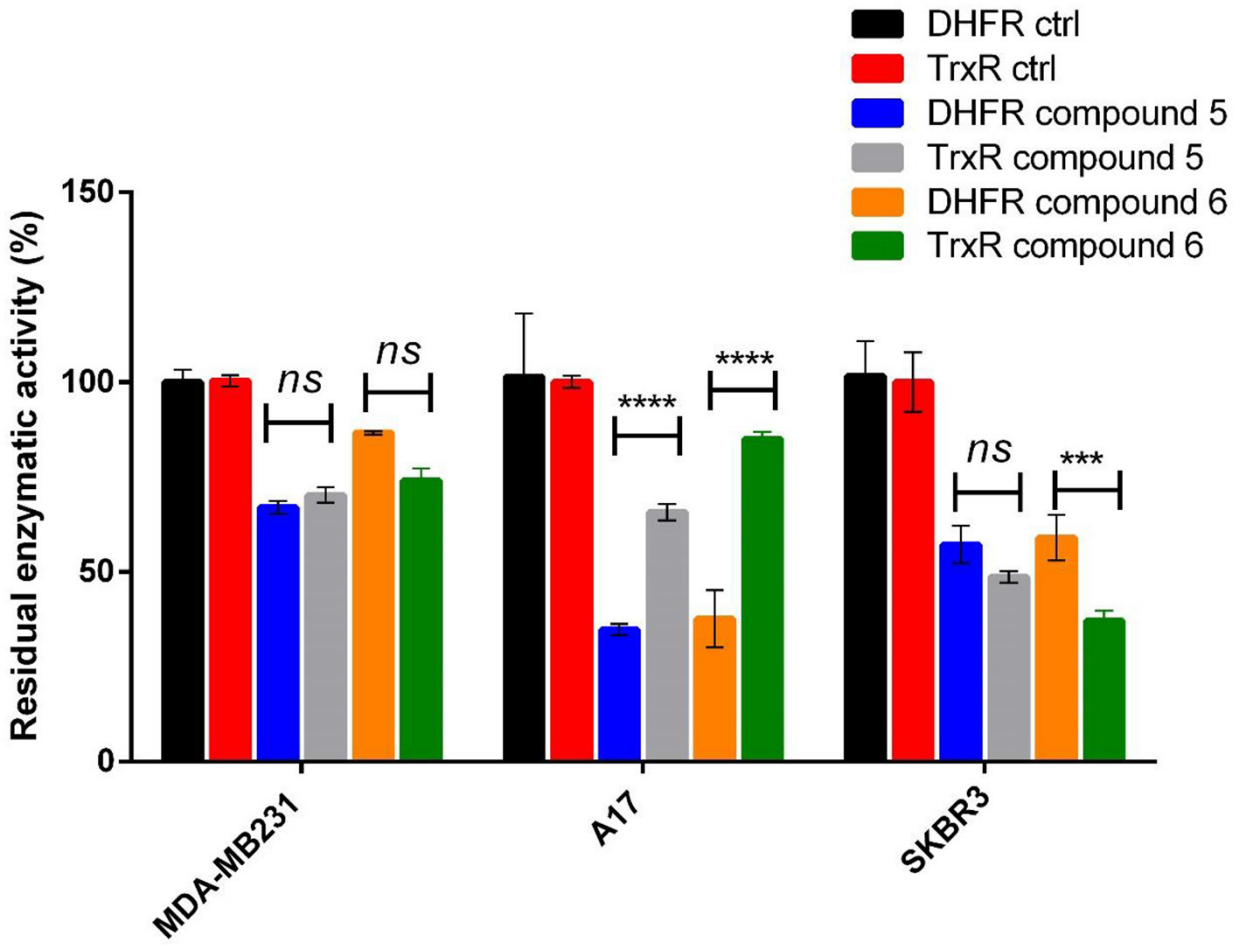
Residual DHFR and TrxR enzymatic activity values measured in breast cancer cell lines after treatment with gold compounds **5**, 4,5-dicyano-imidazolate-1yl-gold(I)-(triphenylphosphane), and **6**, 4,5-dichloro-imidazolate-1yl-gold(I)-(triphenylphosphane), at 12 h of treatment. The reported percentage residual activities are relative to the concentration of gold(I) compounds nearest to the IC50 obtained by MTT assays: 8 μM of compound **5** and 4 μM of compound **6** for MDA-MB23, 12 μM of both compounds for A17, 6 μM of both compounds for SKBR3 cells. The statistical significance relative to the difference between residual DHFR and TrxR enzymatic activity in each cell line is reported: ****p< *0.0001*, ***p< *0.001, n.s. not significant*.

The interaction of the gold(I) complexes with ct-DNA was determined by analyzing the competitive EtBr displacement according to fluorometric studies. Figure 6S reports the emission spectra of the EB-ct-DNA in buffer (10 mM Tris-HCl buffer pH 7.4, 10 mM NaCl) upon further addition of amounts of the tested gold(I) complexes. The addition of different concentrations of compounds **5** or **6** (5 mM in DMSO), to the EB-bound ct-DNA solution caused modest reduction in emission intensity, indicating that these compounds are able to substitute the EB in the ct-DNA-bound EB and to bind ct-DNA. The quenching behavior quantitatively related to the magnitude of the binding strength of gold(I) complexes, has been then analyzed by the Stern–Volmer equation (Equation 3, material and methods) and values of the quenching constants calculated, K_sv_, were obtained as a slope from the plot of F_0_/F vs. [C] (Figure 4S) and the K_sv_ values are reported in Table 5. Our findings confirmed that both compounds **5** and **6** bind to the EB-ctDNA with low affinity and only little differences were observed as shown from the titration experiments.

#### Competitive Binding Fluorescence Studies With DAPI

As shown in Figure 4, the addition of compound **5** or compound **6** to the DAPI-ct-DNA complex (in 10 mM Tris-HCl buffer pH 7.4 10 mM NaCl) reduces its fluorescence intensity (Zaitsev and Kowalczykowski, 1998), clearly suggesting that both gold compounds show binding affinity to adenine-thymine rich region of DNA and are able to display DAPI from the biopolymer. Data were analyzed by the Stern–Volmer equation (Equation 3, materials and methods) and values of the quenching constants calculated, K_sv_, were evaluated for compounds **5** and **6** as previously described and reported in Table 5. Data revealed an increase in the value of the affinity of the tested compounds for DNA with respect to those reported with the two previous assays without differences between the two gold(I) complexes. The results are consistent with that of absorption spectroscopic studies and approximately indicate a major preference of these gold(I) complexes to bind on the minor groove of DNA.

### Protein Binding Studies

#### Fluorescence Studies on BSA

The study of the interaction of gold-drugs with BSA affords information on the distribution, on the free concentration, on the metabolism and on the efficacy of the gold compound (Radisavljević et al., 2019). The BSA protein has large homology with Human Serum Albumin (HSA) and the quenching of the intrinsic tryptophan fluorescence represents a good tool to deeply investigate the possible interaction of our metallodrugs with BSA in an attempt to characterize the fate of the gold compounds once they are in physiological environments. Fluorescence quenching experiments have been carried out by monitoring the emission of tryptophan at 341 nm upon excitation at 285 nm and increasing concentrations of compounds **5** and **6** with BSA (Quinlan et al., 2005). The fluorescence emission spectra of BSA after the interaction with gold complexes to the protein are shown in the supplemental material, Figure 7S. The addition of the complexes to BSA results in a large reduction in the fluorescence intensity without any shift of the emission maximum and the Stern-Volmer binding constants K_sv_ to BSA for all evaluated complexes (Table 5) showing that all complexes bind BSA with good affinity and with a similar value of K_sv_.

#### Fluorescence Quenching Studies of Transferrin

Transferrin has been looked to as a possible transport vehicle for metal-based drugs because of its abundance in the plasma and its ability to bind ferric ions (Worwood, 2002). Most trivalent metals, and a few divalent metals bind specifically to the iron-binding sites of the apoprotein (O'Hara and Koenig, 1986). Fluorimetric titration of human ATF with gold complexes **5** and **6** demonstrated a similar interaction to that with BSA. As an example, Figure 5, shows the titration spectra of ATF with increasing amounts of complexes. Tryptophan fluorescence emission was monitored at 330 nm for complexes The Stern-Volmer plots are shown in the inset images of Figure 5. The values of Ksv collected in Table 5 were obtained from the initial linear section of the Stern-Volmer plots. As in the case of BSA, the Ksv values suggest good affinity of the two gold complexes with values similar to those reported for BSA and with no differences in binding properties between the two complexes.

## Discussion

Five gold(I) compounds containing the N-Au-P structural frames consisting of imidazole or pyrazole and triphenylphosphane moieties in addition to one compound with the structural frame P-Au-Cl, the 2-benzoic-diphenylphosphanegold chloride, were synthesized to test their cytotoxic activity on BC cell lines to verify an overall activity for these gold complexes for BC's subtypes and sketch a correlation between the structure and the anticancer activity. The compounds are featured by molecular backbones containing a polar azole and a lipophilic triphenylphosphane head, whose polarity and skill to bind was enhanced by the introduction of hydrophilic moieties such as COOH and/or OH groups (Santini et al., 2011). The BC SKBR3 cells display an epithelial morphology in tissue culture and are a useful preclinical model to screen for new therapeutic agents which could overcome the drawback of resistance to HER2-targeted therapies, therefore these cells were considered for MTT tests. The results, reported in Figure 2 and Figure 1S, showed a trend of activity depending on the structure of the gold compounds; in fact, the most active compounds are the compounds **5** and **6**, while the compounds **1**, **2** and **3**, possessing the –COOH functional group on the PPh_3_ moiety or the CH_2_OH group on the imidazole, did not show relevant cytotoxic activity. Therefore, from the MTT assays screening on SKBR3 cells we can conclude that the presence of ligands with hydrogen bonding donor sites in the gold(I) compounds introduces in some way an impeding for the *in vitro* activity. After the screening on the HER2 overexpressing SKBR3 BC cells, the most active compounds **5** and **6** were chosen for other BC subtype cell models: the MDA-MB231 and the A17 (Figure 2S). In addition to compounds **5** and **6**, compound **4** was tested to verify the effect of a different azole, the 3,5-dinitropyrazole, resulting all strongly active with IC_50_ values depending on the cell phenotype. The analysis of the data (see Table 3) reveals an influence of the azolate ligands on the mechanism of action even though they result do not be cytotoxic if tested as free ligands. In particular, compound **4** is the fastest to reach the plateau of effect with all the cell lines, while as concerns compound **5** and **6**, the latter is the most effective supporting the attribution of a presumed role to the substituents on the imidazole. Remarkably, the treatment of human breast cancer MDA-MB231 cells, which is the panel corresponding to the worst diagnosis, show encouraging IC_50_ values, albeit they are in the micromolar range. Unfortunately, even though it was found to be cytotoxic and rapidly active, compound **4** was less stable in physiological condition, likely for the presence of reactive nitro groups. After these cytotoxic studies and their results, further studies were led only with compounds **5** and **6**.

The cancer cells treated with the gold(I) compounds **5** and **6** were then used for enzymatic tests with the aim to determine the DHFR or the TrxR specific activities. The results are shown in Tables 2 and 3. While TrxR is fully recognized as the most likely target for gold(I) compounds, the former was serendipitously found to be inhibited *in vitro* by gold(I) phosphane compounds with IC_50_ in the micromolar range (Galassi et al., 2015). The DHFR specific activity for the three cancer cell lines are noticeably different, underlining that the inhibition was remarkable for the SKBR3, the A17 and MDA-MB231 cells and featuring, in some way, the influence of the cell phenotype. Noteworthy, both gold(I) compounds exerted mild or not significant inhibitory action on the regard of DHFR of healthy NIH-3T3 cells (data not shown). Interestingly, comparing the DHFR's specific activity of the cells treated with gold compounds with those of the cells used as the control, as shown in Figure 3, it is evident that the DHFR's specific activity is differently affected by the gold based compound depending on the time of treatment. These results highlight the role of time in the inhibitory effect exerted by gold compounds toward this target enzyme. The diverse DHFR specific activities herein obtained may derive from the different levels of expression of the enzyme due to variances in cell cycle regulation. Being required for DNA synthesis, DHFR expression is transcriptionally regulated during the growth/cell cycle, with the highest expression levels occurring in the late G1 to early S phase following stimulation of quiescent (G0) cells (Jensen et al., 1997). The different specific activity of DHFR in non-treated MDA-MB231 and A17 cells at 4 h with respect to 12 h, could reflect a phenotypic response to the regulation of the G1 checkpoint and S-phase progression in these two cell lines.

As concerns the enzyme TrxR, the system TrxR/Trx is responsible for the reduction of redox-sensitive proteins upon oxidative stress and the TrxR/Trx expression is augmented in a variety of human malignancies, including lung, colorectal, cervical, hepatic, and pancreatic cancer (Lincoln et al., 2003). Therefore, both DHFR and TrxR are enzymes involved in cell proliferation and cancer progression, and they represent suitable targets for multiple ligands approach in anticancer drug design (Hui-Li et al., 2016); moreover, the use of drugs with different biological targets can be a strategy in overcoming drug resistance. Compounds **5** and **6** exert this multi-targeted inhibitory activity with respect to both TrxR and DHFR. The presence of the soft metal gold(I) ion, confers selectivity toward the selenocysteine residue in the TrxR active site (Galassi et al., 2012), while the hydrophobic phosphane group and the amphipathic azolate moiety can easily fit in the DHFR's substrate binding pocket. In Figure 6, we report the comparison between the residual enzymatic activities of DHFR and TrxR in SKBR3, MDA-MB231 and A17 cell lines in presence of compounds **5** and **6** at the IC_50_ concentrations. While in the MDA-MB231 cells the two enzymes do not show significant differences in their response to both gold compounds, in the A17 cells DHFR activity undergoes a stronger decrease compared to TrxR; these effects were found for both compounds. As concern the SKBR3 cells, it is possible to observe a different behavior of the two gold(I) compounds, while compound **5** displays similar effects for both enzymes, compound **6** inhibits more strongly the TrxR. The different behavior of the gold(I) based complexes toward DHFR and TrxR enzymatic levels in the breast cancer cell lines MDA-MB231 and A17 can reflect either the different mechanism of action of the two compounds in interacting with DHFR and TrxR, and/or the differences in their impact on the two enzymes expression levels in cancer cells. While it is well-recognized that gold(I) complexes can modify the accessible selenocysteine residue in the active site of TrxR (Bindoli et al., 2009), the mechanism by which these drugs inhibit DHFR, seems to be independent by “auration” or oxidation of Cys residues, and rather to be based on reversible competition with the substrate in binding to the active site (Galassi et al., 2015).

It is mostly recognized that enzymes and proteins are preferred binding sites for Au(I) phosphane compounds once in a biological environment, whereas nucleic acids have been little investigated. In this regard, studies have been carried out on the effective interaction with the calf thymus DNA, ct-DNA. The data obtained show a poor aptitude for interaction with DNA, with binding constants of 1.46 mM^−1^ for compound **5** and 3.44 mM^−1^for compound **6** (Table 5). These values are similar to those reported for other gold complexes (Nobili et al., 2010) but lower with respect to those observed for typical classical intercalators, as ethidium bromide, acridine orange and methylene blue showing binding constants of K_EB_ = 6.58 × 10^4^ M^−1^, K_AO_ = 2.69 × 10^4^ M^−1^ and K_MB_ = 2.13 × 10^4^ M^−1^, respectively (Nafisi et al., 2007). The study carried out on the competitive displacement with classic DNA's intercalators such as ethidium bromide and DAPI gave an additional insight; as expected, the K_sv_ values calculated by the emission quenching experiments highlight that gold(I) compounds mildly compete with both intercalators with a major ability to displace the DAPI, showing that a left shifted equilibrium of interaction occurs in the minor groove of the DNA.

Lastly, the reactivity of gold(I) compounds with serum target proteins such as bovine serum albumin and apo transferrin has been investigated. The quenching of spontaneous emissions of these proteins upon addition of increasing concentration of gold(I) phosphane compounds have been detected in both cases; data were processed according to the Stern Volmer model of interaction having as results the values of the binding constants, which are resumed in Table 5. The data show high affinity for both proteins with similar K_sv_ values consisting of few unit × 10^4^ M^−1^. BSA has been already identified as a likely transporter for gold moieties, while the transferrin has never been considered, even though it represents the preferred transporter for metal ions (Gkouvatsos et al., 2012). The biological activity of ATF is strictly linked to its interactions with transferrin receptors (TfR) that regulates cellular uptake; TfRs are upregulated in the surface of cancer cells and thus can be used as selective target in anticancer drug design (Wang et al., 2000; Daniels et al., 2012). Moreover, TfRs are efficient systems to internalize antitumor compounds through the carrier protein ATF directly into cancer cells, circumventing adverse effects against healthy cells receptors (Singh et al., 1998). Our results do encourage further studies to clarify the mechanism involved in ATF binding of compounds **5** and **6** and verify the ability to release these complexes in an active form.

## Conclusions

A class of azolate/phosphane gold(I) compounds has been evaluated for a screening on the regards of BC cell panels. Among the six candidates, only the compounds having the P-Au-N environment and neither hydroxyl nor carboxyl groups in the ligands were found active. Hence, the presence of polar protic functional groups hampers in some way the cytotoxic activity. The most effective compounds are the 4,5-(CN)_2_-imidazolate (compound **5**) and the 4,5-Cl_2_-imidazolate (compound **6**) which were already found to be very active against BLBC *in vivo* (Gambini et al., 2018). Therefore, the present study provides further evidence of the efficacy of these two imidazolate phosphane gold(I) compounds *in vitro* as anticancer even against another BC's subtype, the HER2-positive breast cancer. The two compounds, even though they are very similar in their structure, show MTT and biochemical assays experimental results which are quite different, evidencing a sensitive relationship between the structure and the anticancer activity. For example, the determination of the residual activity of target enzymes in the treated cancer cells reveal the ability of these gold compounds **5** and **6** to strongly inhibit TrxR and DHFR. In fact, the enzymatic assays on the lysates of the treated cells of different BC subtypes showed the strong inhibition of TrxR seleno-enzyme and, surprisingly, of another selected enzyme such as DHFR in an extent not so much dissimilar, despite the fact that inhibition tests *in vitro* on the activity of these enzymes afforded to values of Ki different up to an order of 10^3^ (Galassi et al., 2012, 2015). Remarkably, the enzymatic study on the cells' lysates underlined that the DHFR inhibition effects were observed to be dependent on treatment's time, likely as a consequence of the different cancer cells cycle regulation and the enzyme's expression levels (Figure 6). In this work, it was also highlighted that these compounds strongly bind plasma proteins as BSA and ATF, suggesting their possible role as transport vehicles for these metal-based drugs once administered *in vivo*. Conversely, as expected, they do not display any strong binding to ct-DNA, though they exhibit weak interactions with the minor groove of the biopolymer. On conclusion, two of the herein considered gold compounds confirm their strong anticancer activities *in vitro* on the regards of aggressive BC subtypes cells through multitarget mechanisms: it was assessed their action through the inhibition of both TrxR and DHFR enzymes and their binding to transporter proteins as BSA and ATF, that *in vivo* raised much attention for their possible involvement in the mechanism of action.

## Data Availability Statement

The original contributions presented in the study are included in the article/Supplementary Materials, further inquiries can be directed to the corresponding author/s.

## Author Contributions

VG and JW have performed the MTT tests. CM and AA have funded the cellular studies and elaborated the MTT data. GL has performed and made the interpretation on the protein binding studies and the Thioredoxin Reductase inhibition. SP has performed the study on the DHFR enzyme inhibition and made the interpretation of the data. SV has funded the DHFR studies and supervised the study on the DHFR. LL has prepared the gold compounds and followed some of the enzymatic studies. RG funded and supervised the synthesis of the compounds and wrote the manuscript. All the authors took part at the rielaboration of the data.

## Conflict of Interest

The authors declare that the research was conducted in the absence of any commercial or financial relationships that could be construed as a potential conflict of interest.
