# Supplementary material for: Multi-Targeted Anticancer Activity of Imidazolate Phosphane Gold(I) Compounds by Inhibition of DHFR and TrxR in Breast Cancer Cells
Source: Front Chem. 2021 Jan 11;8:602845. doi: 10.3389/fchem.2020.602845 (PMC7821381; doi:10.3389/fchem.2020.602845)
Supplement: Supplementary file 1 [file Table_1.doc]

**SUPPLEMENTARY data**

**List of contents:**

1. **MTT tests**

**1.1 72 h MTT assays on SKBR3 cells**

1. **In vitro inhibition of purified TrxR in cancer cells**
2. **SKBR3 cancer cells images**
3. **Fluorescence studies**

**4.1 Binding constant determination**

**4.2. BSA binding**

**1.MTT tests**


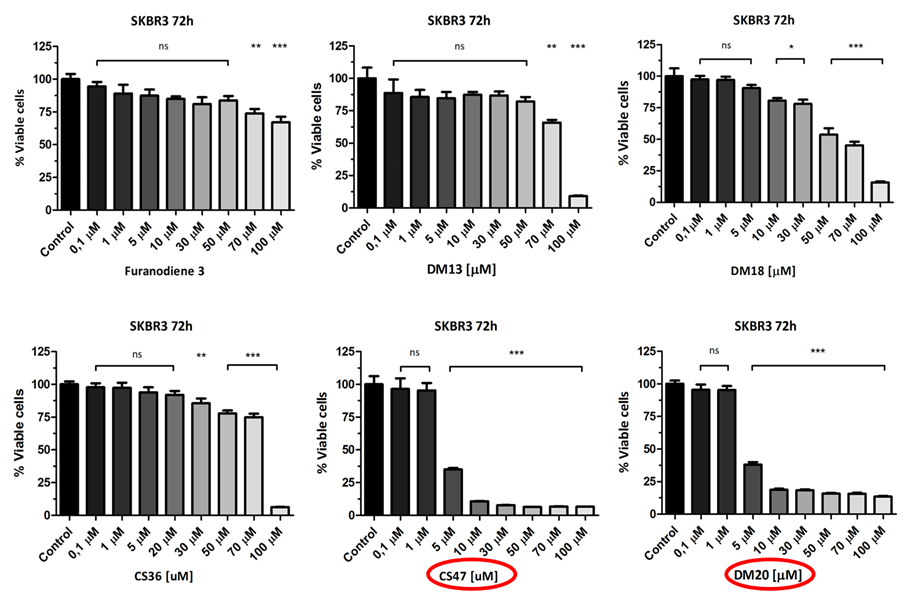


Compound 1

Compound 2


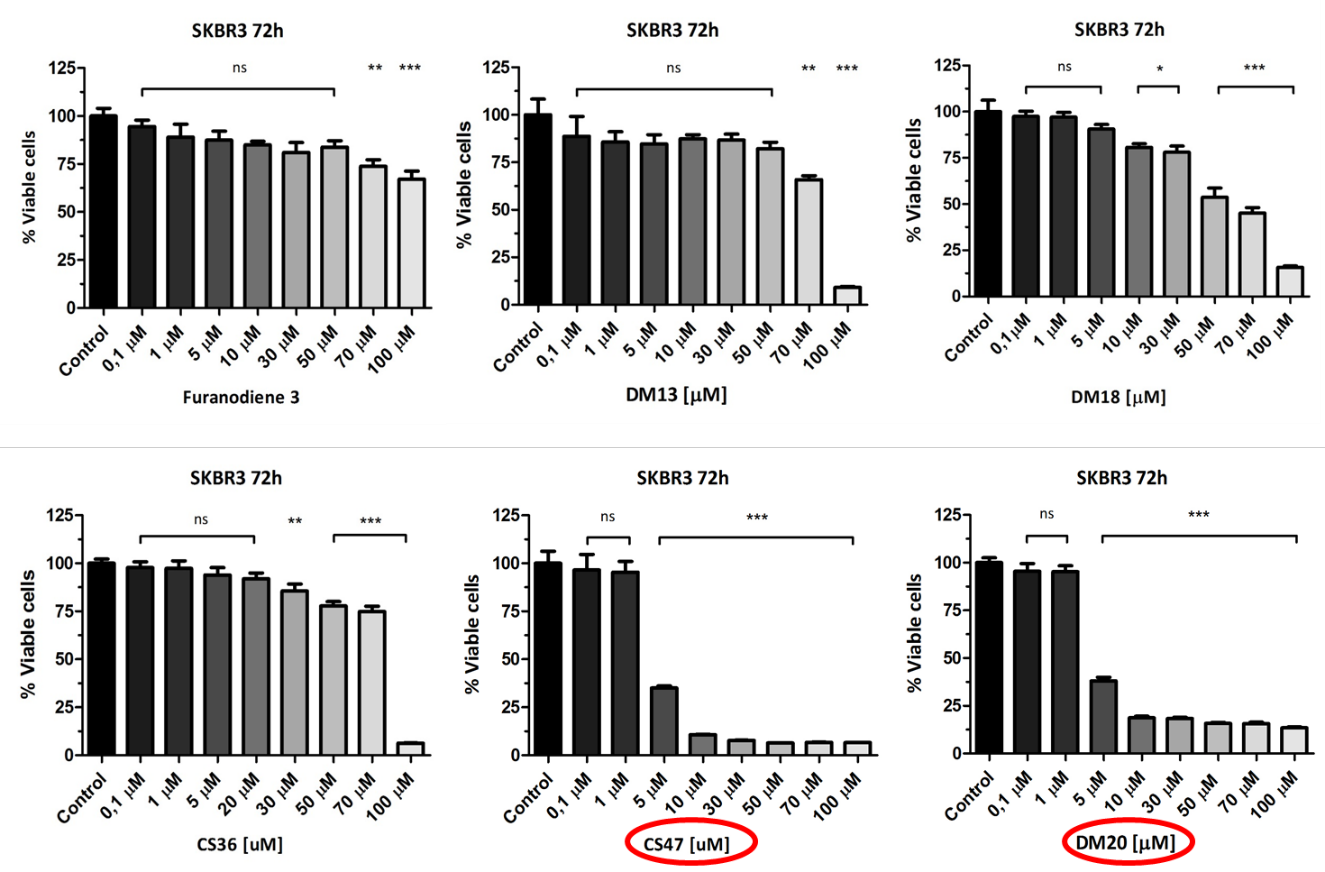


Compound 3

Compound 6

Compound 5

Figure 1S. SKBR3 cells were incubated for 72 hours in the presence of vehicle (DMSO) or increasing concentrations of Compounds **1**, **2**, **3**, **5**, **6** and cell viability was determined by MTT assay. The results are expressed as percentage of living cells with respect to control (vehicle alone). Columns, mean of three separate experiments wherein each treatment was repeated in 8 wells; bars, Standard Error. Statistics: One-way ANOVA followed by Dunnett's multiple comparisons test.


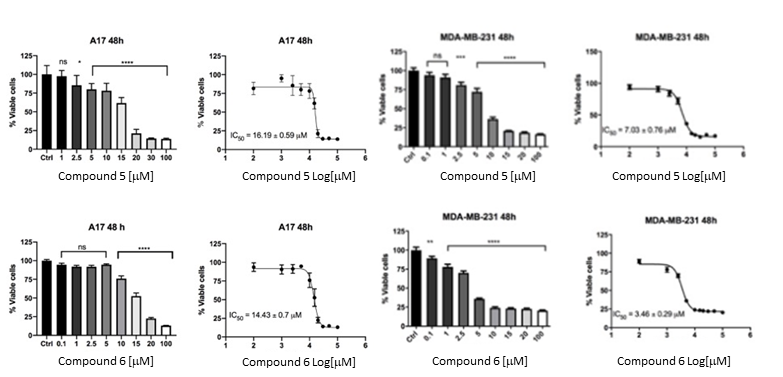


Figure 2S. A17 ana MDA-MB231 cells were incubated for 48 hours in the presence of vehicle (DMSO) or increasing concentrations of Compounds **5** and **6** and cell viability was determined by MTT assay. The results are expressed as percentage of living cells with respect to control (vehicle alone). Columns, mean of three separate experiments wherein each treatment was repeated in 8 wells; bars, Standard Error. Statistics: One-way ANOVA followed by Dunnett's multiple comparisons test.

IC_50_ values were calculated for each of the cell lines tested, by fitting the concentration-effect curves data obtained in the three experiments with the sigmoid-Emax model using nonlinear regression, weighted by the reciprocal of the square of the predicted effect.

**2.In vitro inhibition of purified TrxR in cancer cells**

The inhibitory effects of gold(I) complexes on total Thioredoxine reductase (TrxR) was measured according to standard procedures and tested at increasing concentrations near the IC_50_ values calculated taken in account the date from the dose effect curves (Table 2 in main manuscript).


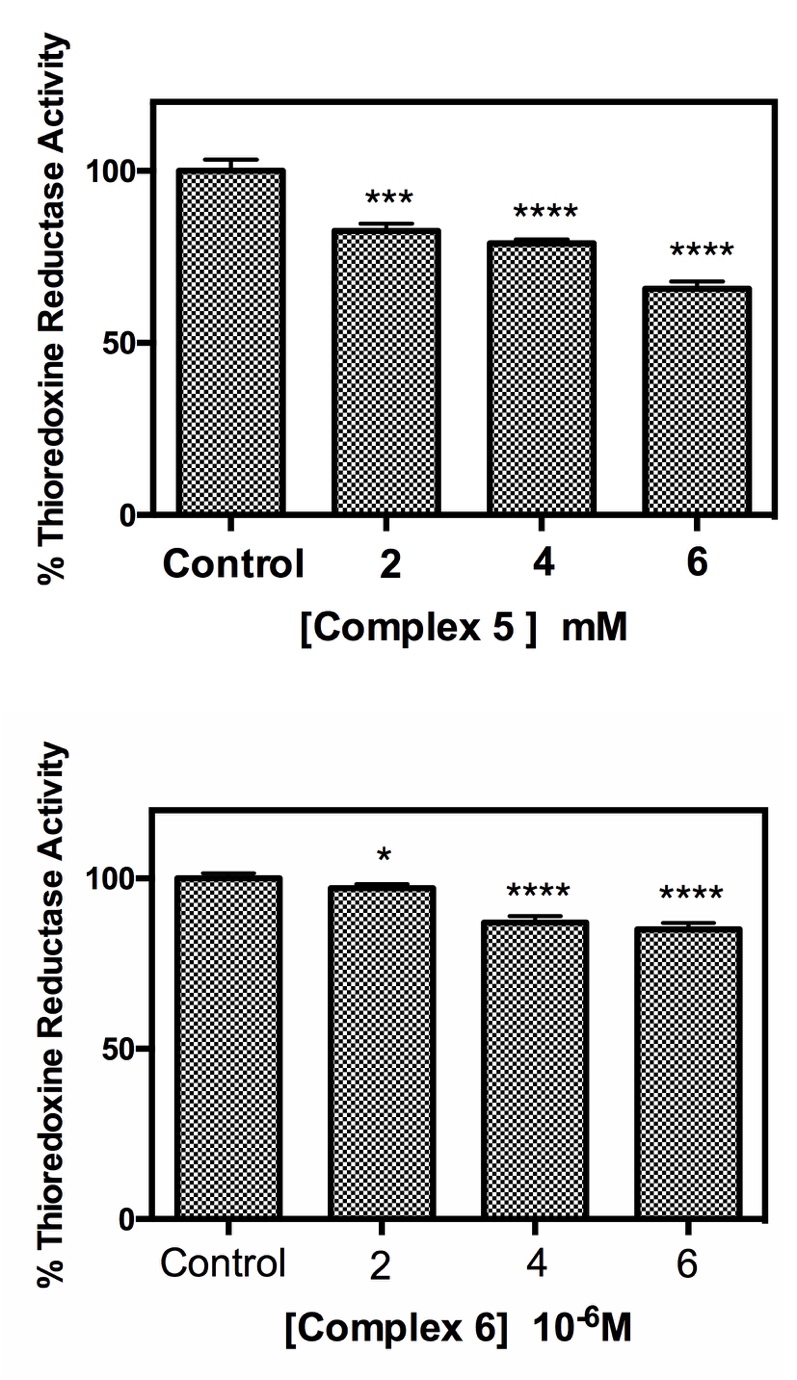


Fig. 3S. Effects of gold(I) compounds 5 and 6 on redox enzyme thioredoxine reductase in A17 cancer cells. Cells were incubated for 12 hours with different amount of tested compounds 5 and 6. After incubation, cells were washed twice with PBS and lysed. TrxR activity was tested by measuring NADPH-dependent reduction of DTNB (Galassi et al, 2012).


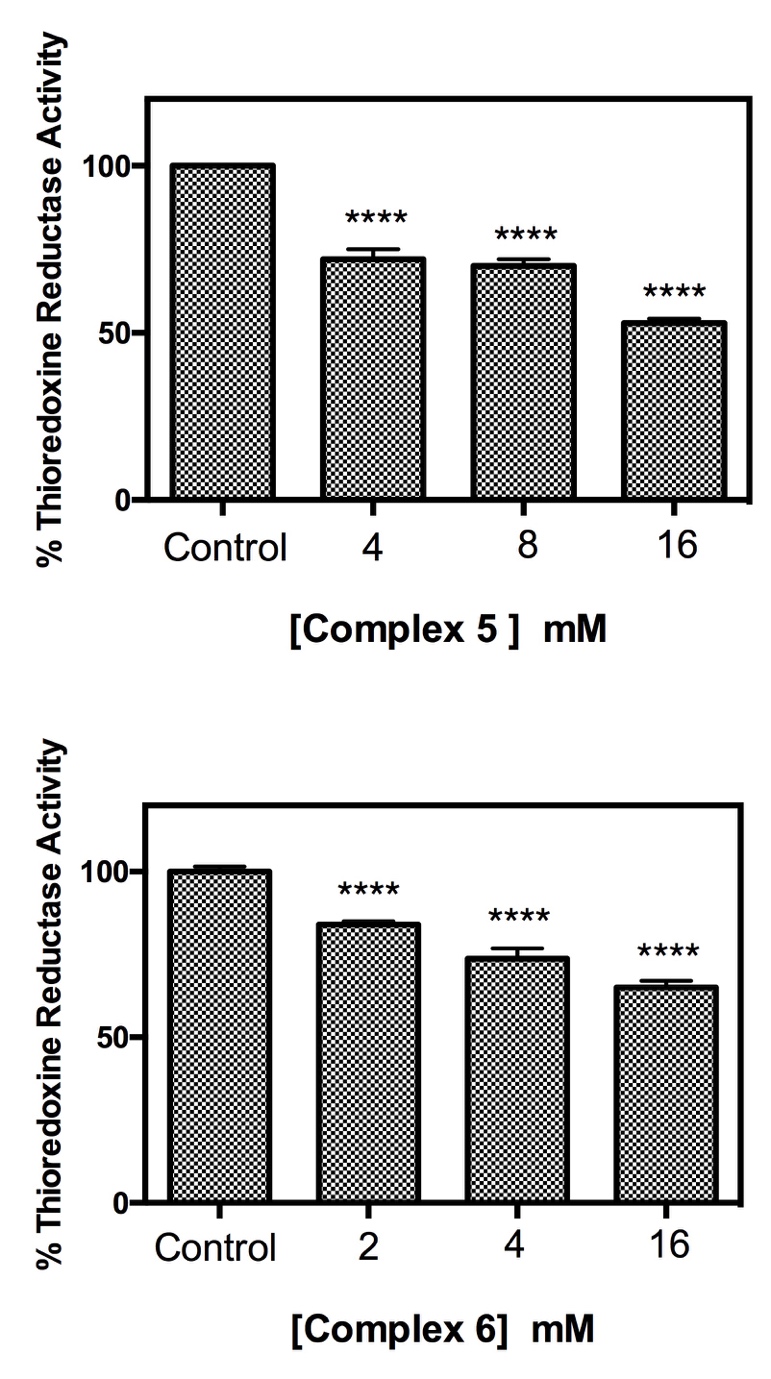


Fig. 4S. Effects of gold(I) compounds 5 and 6 on redox enzyme thioredoxine reductase in MDA-MB231 cancer cells. The cells were incubated for 12 hours with different amount of tested compounds 5 and 6. After incubation, cells were washed twice with PBS and lysed. TrxR activity was tested by measuring NADPH-dependent reduction of DTNB (Galassi et al, 2012).

**3.SKBR3 cancer cells images**

Following we report some representative pictures of cells treated with compound 5 and 6 at the indicated concentration for 4 or 12 hours just before to take cell lysates for the enzymatic tests.


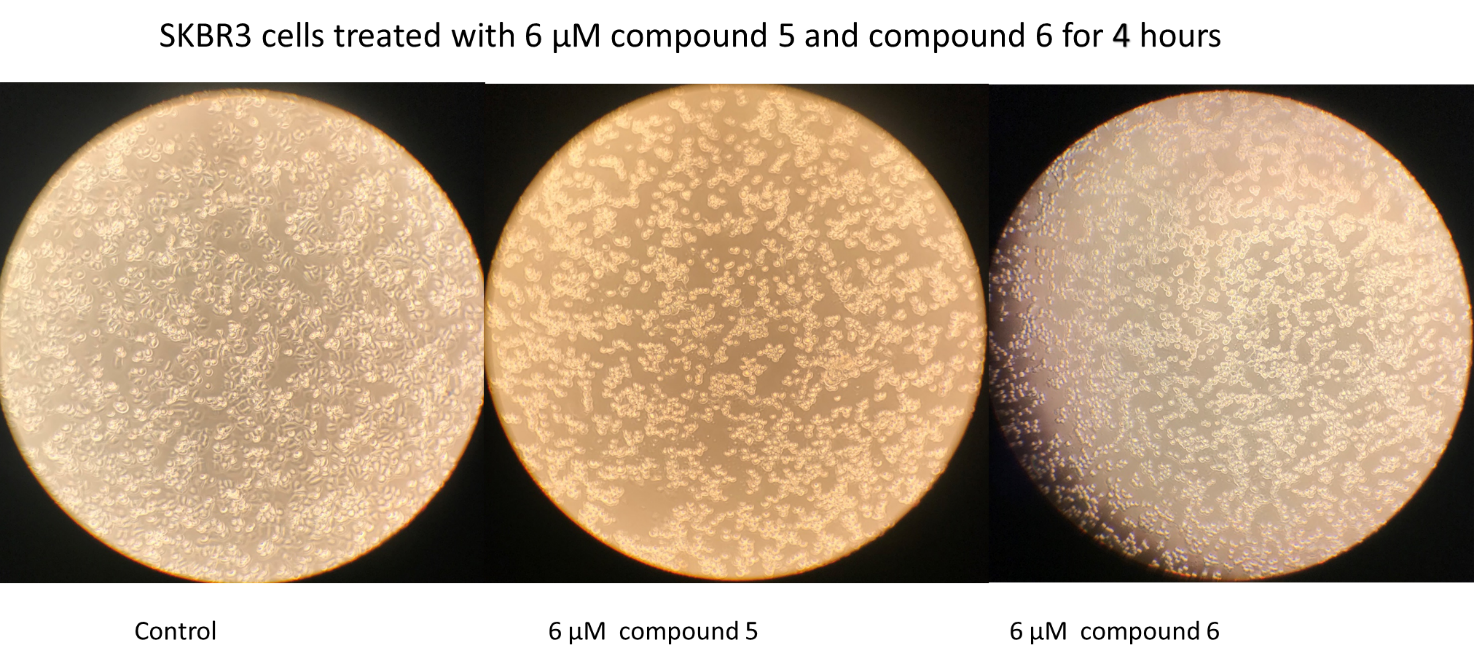


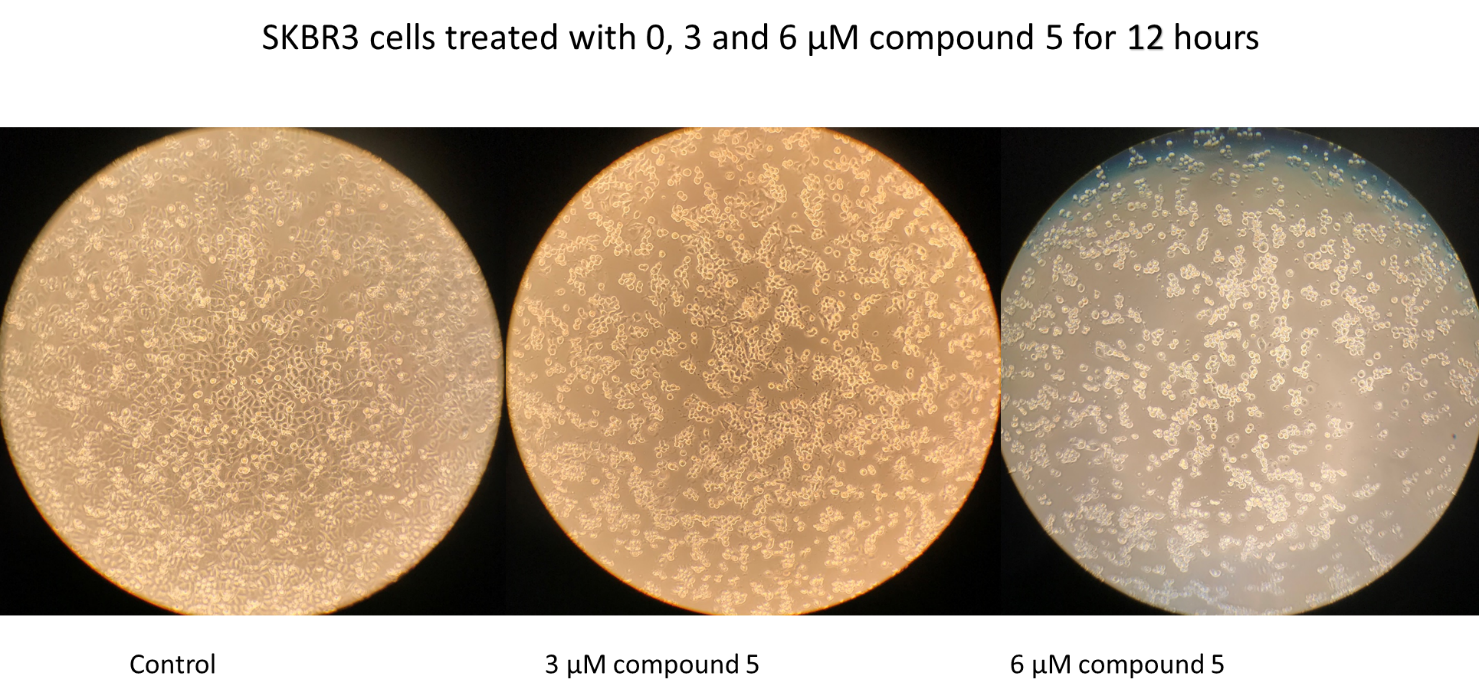


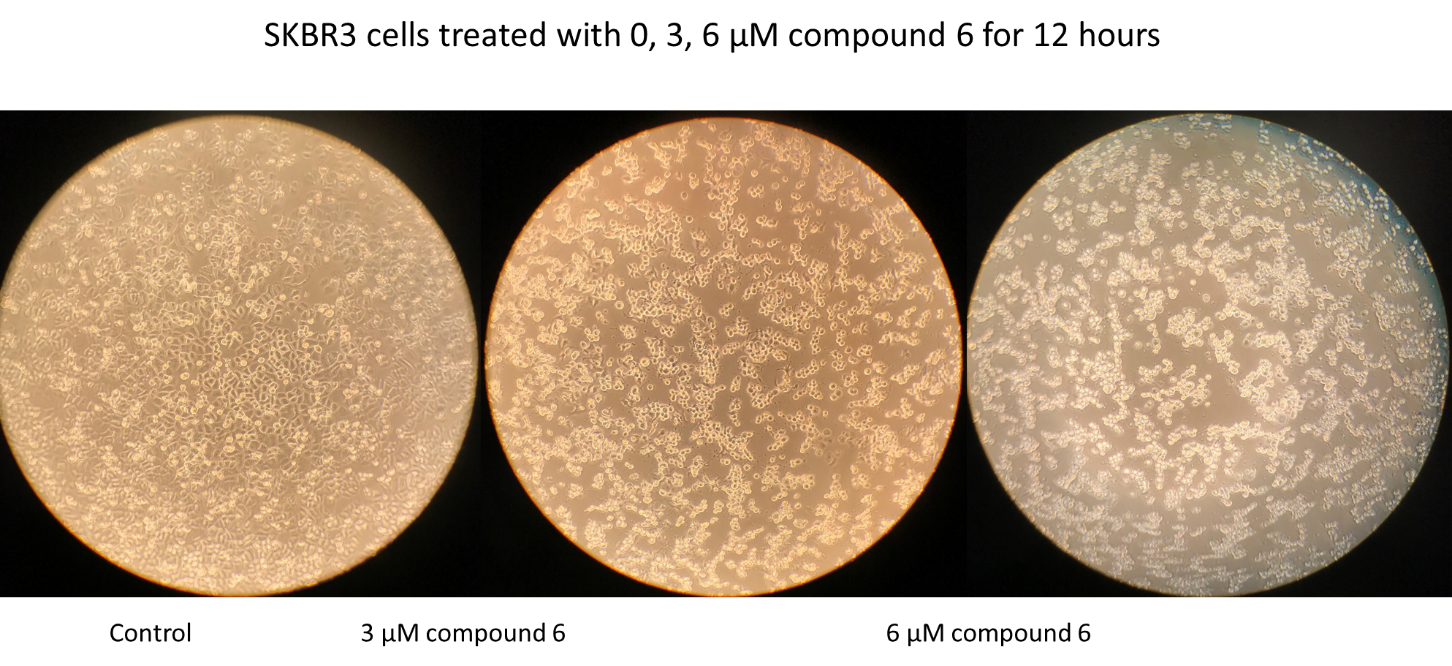


**4.Fluorescence studies**

**4.1 Binding constant determination**

For the different compounds the binding constant (Kb) was determined from the spectroscopic titration data using the following equation

Eq. (1) [DNA]/(ɛa - ɛf)=[DNA]/( ɛb - ɛf) + 1/Kb(ɛb -ɛf),

where [DNA] is the concentration of DNA in base pairs, the apparent absorption coefficient (ɛa) was obtained by calculating A_obsd_/[compound]. The terms ɛf and ɛb correspond to the extinction coefficient of free (unbound) and the fully bound compound, respectively. A plot of [DNA]/(ɛa - ɛf) versus [DNA] will give a slope 1/(ɛb - ɛf) and an intercept 1/Kb(ɛb - ɛf). Kb is given by the ratio of the slope to the intercept.


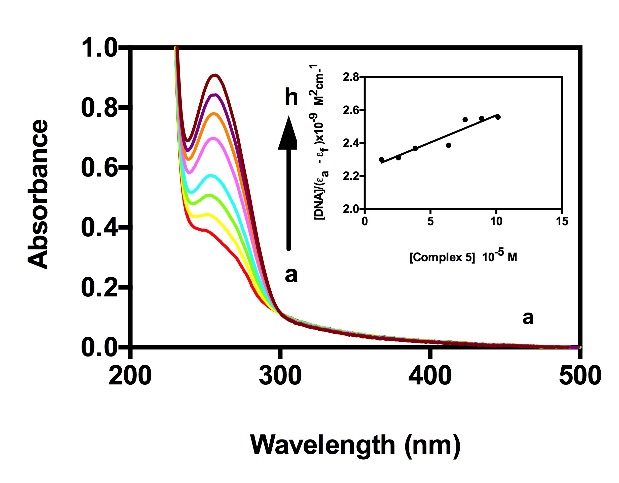

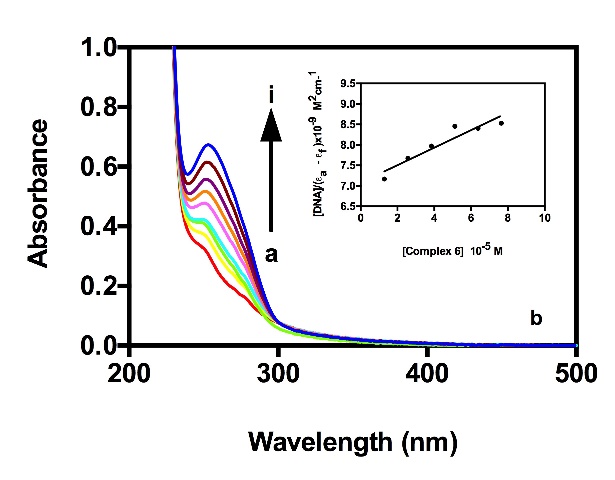


1. b)

Figure 5S. The UV spectra of compound **5** (a) and **6** (b) and addition of ct-DNA. The arrow indicates the changes of the bands upon addition of ct-DNA. Plot of [DNA]/(ta – tf) versus [DNA] for the titration of DNA; solid line is linear fitting of the data following Equation 1.

**4.1.a Competitive binding experiments**

Competitive binding experiments were performed maintaining the ethidium bromide (EB) and ct-DNA concentration at 5 μM and 55.7 μM, respectively, while increasing concentrations of compounds **5** and **6** were added to the buffer solution (10 mM Tris–HCl pH 7.4, 10mM NaCl). Fluorescence quenching spectra were recorded using a Hitachi 4500 spectrofluorimeter with an excitation wavelength of 490 nm and emission spectrum 500-700 nm. The fluorescence spectra were recorded and the fluorescence value of decrease in emission spectra were corrected according to the relationship:

Fc = Fm × e (A1+A2)/2 Eq 2

where Fc and Fm are the corrected and measured fluorescence, respectively. A1 and A2 are the absorbance of tested compounds at the exciting and emission wavelengths. For fluorescence quenching experiments, Stern-Volmer's equation was used (Eq. 3):

F0/Fc = 1+ kqτ0 [C] = 1 + Ksv [C] Eq 3

where F0 and Fc represent the fluorescence intensity in the absence and in the presence of the metal complex, [C] is the concentration of the metal complex and Ksv is the Stern-Volmer constant that can be obtained from fluorescence data plotted as F0/Fc vs. the metal complex concentration [C]. All experiments involving ct-DNA were performed in buffer solution (10 mM tris-HCl buffer pH 7.4 10 mM NaCl) at room temperature.


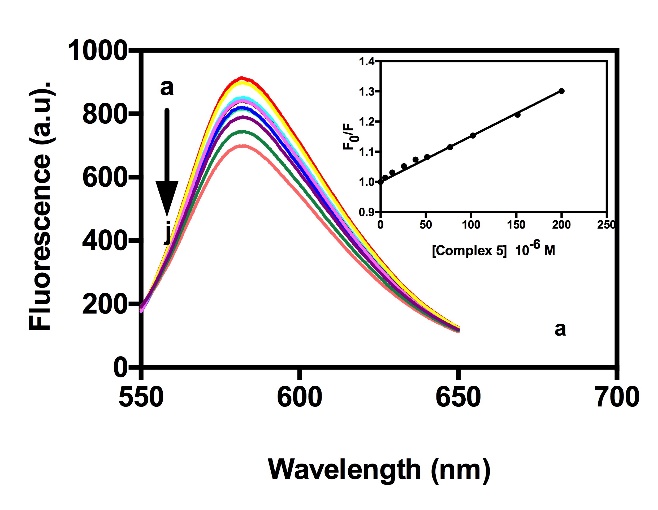

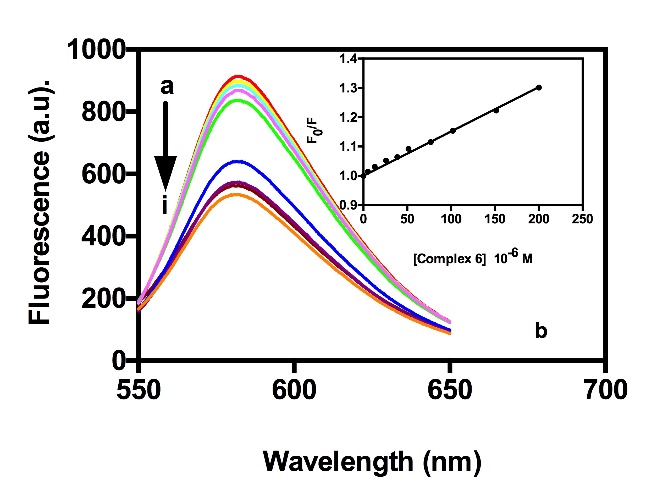


1. b)

Figure 6S. Quenching behaviour in fluorescence of the EB-ctDNA in the presence of different amounts of compound **5** (a) and compound **6** (b). The arrows indicate the changes of the bands upon addition of increasing concentrations of the gold(I) compounds. Stern- Volmer plot of experimental data fitted by Eq. 3.

**4.2. BSA binding**

Protein fluorescence intensity was recorded after each successive addition of complex solution and equilibration (ca. 5 min). Fluorescence spectra were recorded from 300 to 450 nm at an excitation wavelength of 285 nm. The value of Stern Volmer constants of the different metal complexes to BSA were evaluated following the equation 3 and fluorescent values were corrected by equation 2.


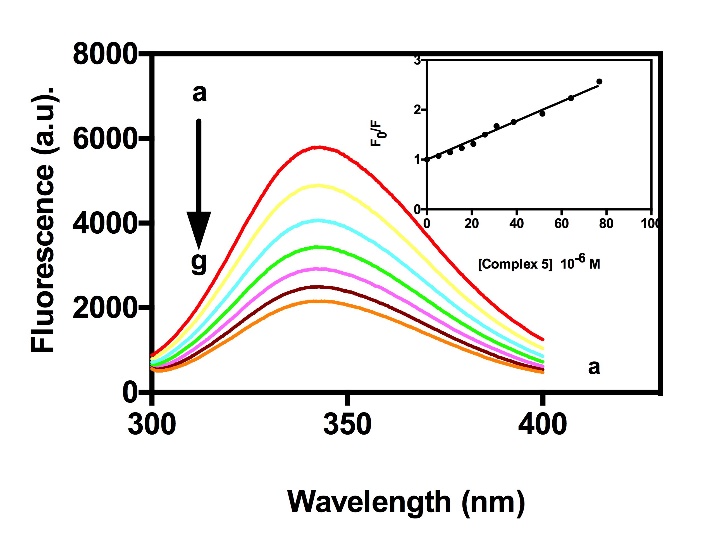

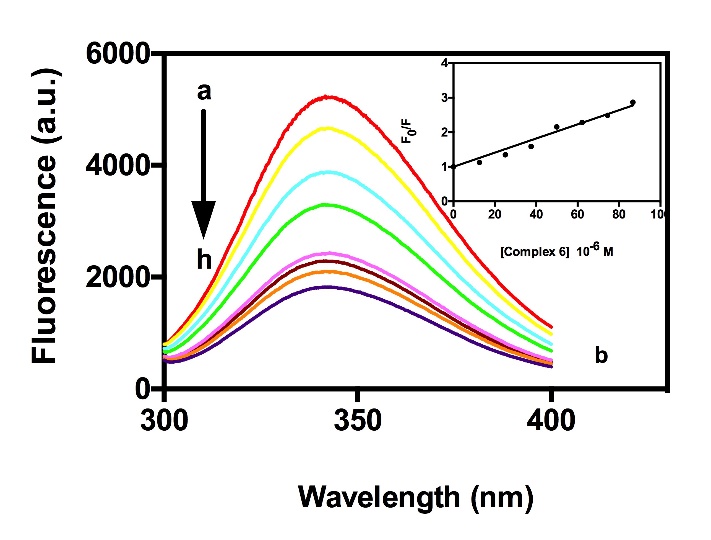


1. b)

Figure 7S. Quenching behaviour in fluorescence of the BSA in the presence of different amounts of compound **5** (a) and compound **6** (b). The arrows indicate the changes of the bands upon addition of increasing concentrations of compound **5** or **6**. Stern - Volmer plot of experimental data were fitted by Eq. 3.

References:

Galassi, R., Burini, A., Ricci, S., Pellei, M., Rigobello, M. P., Citta, A., Dolmella, A., Gandin, V., & Marzano, C. (2012). Synthesis and characterization of azolate gold(I) phosphane complexes as thioredoxin reductase inhibiting antitumor agents. *Dalton Transactions*, *41*(17), 5307–5318. https://doi.org/10.1039/C2DT11781A
